# Part II. Hospital Reports

**Published:** 1827-10

**Authors:** 


### PRACTICAL MEDICINE;

BEING

### The Spirit of the Medical Journals,

*Foreign and Domestic;*

WITH COMMENTARIES.

### PART II.

### HOSPITAL REPORTS.

"Ore trahit quodcunque potest, atque addit acervo."

### 1. DILATATION OF THE URETHRA.

[Hôtel Dieu.]

The treatment of stricture of the urethra by mechanical dilatation is well known and commonly practised, whilst the difficulties which, in certain cases, attend that treatment, have often proved a source of considerable annoyance to surgeons. Any plan which proposes to obviate these inconveniences of course deserves consideration, and for this reason, we shall lay before our readers, without further preface or apology, an abstract of a paper on the subject by Baron Dupuytren.

This celebrated surgeon observes that there are two methods of overcoming a stricture in the urethra. The first consists in the introduction of a fine bougie *into* the stricture, which is acted on by the mere mechanical pressure of the instrument. This M. Dupuytren terms the *mechanical dilatation* of the canal, and is the plan in common use. Two or three cases are cited in illustration, but these it is not necessary to notice.

There is, however, another mode of proceeding, namely, by what our author calls the *dilatation vitale.* The instrument is of larger calibre and blunt at the extremity; as before, it is passed into the urethra, but not *into* the stricture. When it has reached this point it is introduced no farther, but kept in this situation for a certain time. That this is not a distinction without difference, it is stated that, frequently when it has been found impossible to pass a fine bougie, a blunt instrument has been introduced into the urethra, and kept anterior to, and in contact with the stricture for eight or ten hours, at the end of which time it has been withdrawn, and the bougie has reached the bladder without difficulty. This, however, seems to savour a little of the post hoc, ergo propter hoc argument, for at the first introduction, the bougie may have been obstructed by spasm of the canal, which, at the end of so many hours, may have subsided quite independently of the presence of the larger instrument. But we shall pass, at once, to the cases brought forward by M. Dupuytren in support of his practice.

*Case* 1. Eight or ten years ago, M. Dupuytren was called to M. * * *, a man of property, and of a nervous and extremely susceptible disposition. He was tormented with great difficulty of making water, and M. D. proposed the introduction of bougies into the urethra, but the bare thought of a bougie filled the patient with horror, and it was with the utmost difficulty that he consented. Scarcely had the instrument entered the urethra before all his apprehensions were renewed, and though M. Dupuytren contrived to get the instrument as far as the stricture, such was the agitation of the patient, that he durst go no further. He accordingly left the bougie in the urethra, with the intention of completing its introduction in the course of a few hours. On his arrival at the expiration of the time, he found that the patient had made water freely, and now the instrument was passed fairly into the stricture without difficulty; in a few hours more it was passed still deeper, and during the day it was introduced into the bladder. In two or three days, a larger bougie was employed, and at the end of a fortnight, the gentleman voided his urine in a good stream, and without either pain or difficulty.

This, we apprehend, was a case of "spasmodic stricture," and we can conceive no method more calculated to allay spasm and irritation than that pursued by M. Dupuytren, to wit, the gradual introduction of the instrument. The plan in question appeared to that able surgeon to be well calculated for patients of a pusillanimous or irritable temperament, as also for those cases where the symptoms are not so urgent as to require an immediate removal of the obstacle to the passage of the urine.

*Case* 2. Colomb, æt. 36, entered the Hôtel Dieu, Feb. 6th, 1827, with all the symptoms of stricture. The difficulty of making water had commenced seven or eight years ago, and it followed a gleet of ten years standing. *Feb.* 7. A middling-sized sound was introduced as far as the membranous portion of the urethra, where it was stopped by a very firm stricture, into which it could not be made to enter. Up to this point, a bougie was passed, and left in the urethra, but in the course of an hour it was taken out by the patient. In the evening, an attempt was made to introduce it again, but, in consequence of the excessive spasm of the canal, it could be got no farther than the fossa navicularis, and here it was grasped so firmly that considerable force was necessary to withdraw it. *Feb.* 9. A silver sound was employed with similar want of success. Part of a large sound, rounded at its extremity, was now fixed in the fossa navicularis, and at the end of 24 hours, it was replaced by a middling-sized instrument of elastic gum, which was kept in the urethra for twenty days. Three instruments, each larger than the other, were successively introduced, and the patient, on leaving the hospital, could make water freely and in a large stream.

Two more cases are detailed. In both, the strictures were of long-standing, and in both, the introduction of instruments up to the strictured portion, and leaving them there, proved effectual.

Sounds or bougies of elastic gum, having the extremity rounded off and blunt, and of a length proportioned to the depth of the obstacle, are best adapted for this mode of dilatation. After some hours, and in the worst cases, days, either the blunt instrument itself can be passed into the bladder, or the urethra is so far enlarged as to permit the passage of a bougie. There are many advantages attending a slow and gradual dilatation of the urethra. The pain is comparatively trifling, and there is no danger of producing those lacerations and false passages, which occasionally follow a more rapid introduction of instruments. But, besides this, there is a continual disposition in the urethra to contract again, and the quicker the dilatation, the quicker, cæteris paribus, will be the contraction. This disposition in the urethra may be kept under by the introduction of a bougie every ten, twelve, fifteen, or twenty days, as the case may require. The instrument should be retained in the urethra for some time.

In conclusion, we would just observe, that the above plan deserves a trial in those cases of spasmodic stricture, in which the introduction of an instrument into the bladder is impracticable, or exceedingly difficult. We think that it ought to be employed before the caustic bougie; it is a safer, a milder, and, if effectual, a far preferable mode of treatment.—*Repertoire, Tome 3.*

---

### 2. ON RE-UNION OF DIVIDED TENDONS.

[Dr. Fearn, of North Carolina.—Philadelphia Infirmary.]

*Case.* James Lang, aged 54 years, was admitted into the Infirmary of the Philadelphia Almshouse, on the 31st of March, 1826, with his leg and ankle swelled so extensively, as to preclude the possibility of ascertaining the true nature of the injury. He had stepped off the curb-stone into a gutter, three or four feet deep, the preceding evening, lighting on his toes, and thus straining the extensor muscles of the foot. He felt something give way, (but heard no crack or noise) attended by instantaneous pain, and total inability to walk with that toe on the ground. Absolute rest and strict antiphlogistic measures were enjoined, and persevered in for several weeks, when the injury was found to be a rupture of the tendo Achillis. At this time (30th April) Dr. Horner took charge, and applied a bandage and splint. These were continued a few weeks, without any appearance of union in the divided tendon. The ends of the tendon were found to have formed no adhesions to the neighbouring parts, but moved freely from side to side, when the antagonizing muscles were in action or relaxed. The ends could be placed nearly in contact by favourable position and compression of the muscles. Dr. Horner now adopted the plan proposed by Dr. Physic, in cases of ununited fractures, and passed, by means of a seton-needle, a ribbond, three-quarters of an inch broad, through the space intervening between the ends of the ruptured tendon. The leg was again bandaged by the roller as before, and a splint anteriorly. This process was persevered in for 46 days, when the seton was removed. Inflammation had greatly indurated the parts adjacent to the seton, and, when felt through the integuments, gave more the sensation produced by a cartilage or bone, than common inflamed cellular substance. The ends of the tendon could not be distinguished, but were merged in the indurated mass. The splint and bandages were continued three or four weeks longer, the ulcer being dressed with simple cerate, and healed. Up to the 25th July, the patient had been kept perfectly at rest. The splint and bandage were now removed, and the patient allowed to take gentle exercise by walking. *August 20th.* He is now able to walk with ease, and without a stick. *Sept. 22d.* The tendon is now considered as restored to its pristine strength.

Dr. Fearn has made many experiments on animals, with the view of trying the principle acted on so successfully in the above case, and the results, we think, leave no doubt of its applicability.—*Philad. Journal, May,* 1827.

---

**8. CASES ILLUSTRATIVE OF THE ILL CONSEQUENCES WHICH SOMETIMES FOLLOW THE REDUCTION OF DISLOCATIONS. BY M. FLAUBERT.**

[Hôtel Dieu of Rouen.]

M. Flaubert has detailed some cases of this kind in the Répertoire, and, as they are not uninteresting, we shall give a condensed account of them here.

*Case.* 1.—*Dislocation of the Head of the Humerus forwards.* Le Breton, æt. 57, a sturdy sailor, rather given to drinking, fell upon the arm, which was carried forcibly backwards. This was on the 2d March, 1824. A surgeon applied poultices to the shoulder, but these giving no relief to the pain, the patient entered the Hôtel Dieu of Rouen on the 13th.

When seen by M. Leudet, there was swelling of the left shoulder, arm and fore-arm, which latter, with the hand, was rather cold. There was a hollow beneath the acromion, and the head of the humerus was situated under the pectoral muscle. It being evident that there was dislocation of the head of the humerus forwards, M. Leudet proceeded to the reduction. Accordingly, the patient being seated in a high chair, extension was made from the wrist, whilst the counter-extension was effected by a roller, passed under the arm-pit, the ends crossing over the opposite shoulder, and fixed to a staple in the wall. A ball was kept in the hollow of the axilla, to lift up the head of the bone at the instant of reduction. The extension was made by eight intelligent pupils, M. Leudet taking the management of the arm. At the first attempt, the head of the bone was dislodged from its place, and brought into the axilla; the second trial was followed by its complete reduction. An enormous swelling took place, almost immediately, beneath the pectoral muscles. The face became pale, and covered with sweat—the lips livid, and the pulsation in the radial artery ceased. M. Leudet attributed these symptoms to exhaustion, and the swelling he imagined to be *gas*, escaping into the cellular membrane. After a few minutes, the general symptoms disappeared, but the swelling remained, whilst the pain was intolerable. *Compresses dipped in a solution of acetate of lead, to be applied.* 14*th*. The countenance was pale—the pulse small, hard, and frequent. The swelling seemed to have subsided a little, but the limb was cold, and of a purplish colour. In the axilla was a tumour, the pulsations of which were distinct to the eye, though not to the touch. It was now evident that the artery was ruptured, but the state of the parts around precluded any operation for tying the subclavian. On the 17th, phlyctenæ appeared, and on the succeeding days, gangrene became developed. 25*th*. The fingers, elbow, skin of the axilla, and inside of the arm, are in a state of sphacelus; the pulsations of the tumour are more marked. 27*th*. A good deal of hæmorrhage from two openings, situated a little below the arm-pit. Though the bleeding was arrested, the patient expired in the course of an hour.

*Dissection.* Hand and inside of the arm in a state of gangrene. The pectoralis major was almost completely torn across, and its fibres were separated by clots of blood. The upper portion of the short head of the biceps was ruptured also. All the muscles of the arm, shoulder, and outside of the chest, were infiltrated with blood. Between the pectoralis minor and latissimus dorsi, there was a large clot, on removing which, the axillary artery was found to be fairly torn across, a little above the origin of the subscapular. In order to discover the upper end of the vessel, it was necessary to dissect the *subclavian*, which was enlarged, as were the branches which arise from it. The *axillary* artery lay beneath the pectoralis minor, upon the rib, to which it adhered by means of coagulable lymph. This end of the vessel was narrowed, and the thoracic nerves flattened. The second rib was depressed, its periosteum slightly absorbed, and the bone itself a little rough. The head of the humerus was somewhat flattened at the part corresponding to the rib: the capsule was torn—the cartilage rough and ulcerated in parts. The inner margin of the glenoid cavity was fractured.

*Remarks.* It is difficult to imagine how such extensive injury should take place, without a great deal of violence having been used in the attempts at reduction. Surely the extension must have been severe, to rupture the great pectoral muscle and short head of the biceps, to say nothing of the axillary artery. At the same time, it is surprising what force is daily used, and with no bad effect whatever, in reducing dislocations. Would not amputation at the shoulder-joint have been advisable when gangrene was commencing? If ever amputation, during the progress of mortification, promises to be of service, it is in cases like this, where the disease is clearly local, the consequence of a deficient supply of blood to the limb.

*Case 2.—Dislocation of the Humerus into the Axilla.* Madame G. æt. 64, dislocated the humerus by a fall, Nov. 27th, 1823. She was not seen by M. Flaubert till seven weeks after the accident, and as the pains were less, and motion tolerably free, he dissuaded the patient from submitting to any attempts at reduction. Next day, however, she returned, fully determined to have the dislocation reduced if possible. The extension and counter-extension were each made in the usual way, by five men. The first attempt, which lasted seven or eight minutes, was unsuccessful; a second, and shorter one, effected the reduction, during which, the patient felt as though something "gave way" on the inside of the wrist. The patient was now found to be hemiplegiac on the right side. There was no motion, and sensation was very slight, especially in the arm. The eye of that side was half closed, and there was a slight ecchymosis on the foot and lower third of the leg. M. Flaubert, considering the hemiplegia to be dependent on some extravasation within the head, produced by the agitation and violent efforts of the patient during the operation, bled her immediately. Purgatives were given, and rubefacients, with blisters, applied. The symptoms disappeared in part. In three months the leg was free from paralysis, but a degree of numbness and susceptibility to fatigue remained so late as February, 1826, the date of the last visit. The head of the humerus seemed to be drawn forwards, its motion was imperfect, the patient not being able to lift the hand to the mouth. The hand was useless, the thumb being extended, the other fingers semi-bent. The ring and little finger were quite insensible, and the heat of the limb appeared to be diminished.

*Remarks.* We think it extremely probable that the axillary plexus of nerves was injured. The giving way at the inside of the wrist, and the subsequent paralysis of the ring and little fingers, would seem to point out the ulnar nerve as suffering more particularly. Whether the hemiplegiac seizure was merely the result of sympathetic irritation of the brain, or actual lesion of the organ, of course we cannot pretend to say.

*Case 3.—Dislocation of the Head of the Humerus into the Axilla.* F. æt. 70, of a good constitution, met with a fall on the elbow, Nov. 1, 1825 Five weeks afterwards, she entered the Hôtel Dieu of Rouen, presenting all the symptoms of dislocation into the axilla, on the left side. M. F. being misinformed as to the time which had elapsed since the accident, proceeded to the reduction, which was effected on a second attempt. The patient immediately became affected with great constriction of the chest, sense of suffocation, and the face became purple and injected. These symptoms were followed by an emphysematous effusion, stretching from under the clavicle, across the shoulder, to the middle of the back, where it gradually disappeared. The patient was pale, and the pulse weak, with nausea. In the left thigh and leg there was a sensation of cold and great numbness; the least touch upon the thigh caused exquisite pain. The patient was placed in bed, when she fell into a state of syncope for about an hour, on recovering from which, she complained of imperfection of vision, severe head-ache, and loss of motion in the right arm. In the night, the patient was unable to make water; the catheter was introduced, and half a glassful of urine drawn off. Next day, *Dec.* 9, the deltoid was completely paralyzed, allowing the humerus to drop into the axilla as before. The upper and lower extremities of the left side were almost wholly paralyzed also. *Vesp. Catheterism—same quantity of water obtained.* 10*th.* Pain in the back of the head, ears, and nucha; pain also in the left thigh, with formication in that extremity—left arm quite insensible—pulse quick and rather hard—tongue foul—nausea. *Bleeding to eight ounces, frictions and volatile liniment with tincture of cantharides to the left arm.* 11*th.* Pupil sluggish—towards evening much difficulty of respiration. A purgative glyster was given which acted freely, and on the next day she was better. 13*th.* More pain in the left side. 15*th.* The pain prevents her sleeping. A vast slough was now discovered on the sacrum, she became gradually worse, and on the 26th the breathing was stertorous and the pulse irregular. In the course of the day she expired.

*Dissection.* The pectoralis major was bruised by the roller used for counter-extension, indeed, on the inside the fibres were reduced to a kind of reddish-brown bouillie. On the outer border of this muscle was a cavity containing bloody serum. All the nerves of the arm were united in a mass in the axilla by means of condensed cellular tissue, a circumstance apparently owing to the pressure produced by the head of the bone. On approaching the scaleni muscles, the four last nerves which go to form the axillary plexus, namely, the 6th, 7th, and 8th cervical, and 1st dorsal, were found torn from their origin in the spinal marrow! The brain and its membranes were sound. On opening the vertebral canal the dura mater was of a reddish brown. The tunica arachnoides was injected, particularly in the neck. The spinal marrow at this part presented a range of white spots, marking the place from which the roots of the nerves had been torn. The medulla at this point was thicker than natural, and of the consistence of a reddish-brown bouillie, the grey matter not being distinguishable from the white. The filaments were rather red and injected.

*Remarks.* It certainly seems to us that in this case an extraordinary degree of force must have been employed. Perhaps the age of the patient, 70, might have rendered the parts more lacerable than usual, but still we cannot imagine how the axillary nerves could be torn from the spinal marrow, by any moderate degree of extension. We think that in old persons like the above, dislocations, especially if they have existed any time, should be left to themselves. Such patients cannot undergo with impunity the rude handling which is often necessary in effecting reduction, and a weak limb at their age is generally of but little consequence.

*Case* 4. *Dislocation of the Fore-arm backwards.* A woman, æt. 45, of good constitution, presented herself Dec. 9th, 1826, with the above accident, which had taken place 27 days previously, in consequence of a fall from a voiture. The characters of the dislocation were well marked, and had been recognized by her medical attendant, who only applied a liniment to the elbow. The reduction was attempted twice by seven pupils without success. The patient was then bled, and extension being made again, the ulna slipped into its place, whilst the radius which remained behind was easily pressed by the thumb into its situation. At the instant of reduction, a diminution *(étranglement)* of the elbow-joint took place, whilst a projection appeared above and below it. This was accompanied with a sound like that produced by the tearing of parts, and it seemed to those present, that the muscles which surround the articulation, with all the parts excepting the skin, were rent across. A considerable swelling followed, with a disposition to continual fits of syncope, whilst no pulsation could be felt in the radial artery. The pulse, however, returned next day, and the swelling gradually subsided. On the 26th December, she had an attack of pain in the right side of the chest, which was relieved by leeches. On the 4th January she left the hospital, having tolerably free motion of the arm, none in the fore-arm, and but little mobility in the finger.

*Case* 5. *Dislocation of the Humerus into the Axilla.* The patient, M. Heim, met with the accident fifteen days before he applied to our author. The symptoms of dislocation into the axilla of the left side were well marked, and after employing the warm bath for two hours and a half, attempts were made at reduction. Before, however, this could be effected, the operators were obliged to desist, in consequence of the patient's complaining of excessive pain in the wrist, with loss of motion in the hand and fore-arm, and numbness of the whole of the left lower extremity. Considerable constitutional irritation, and a severe attack of fever followed, whilst there took place, after a time, infiltration of the arm and fore-arm near the elbow, which lasted for several months. In February, a slight power of flexion and extension was remarked in the fingers, but it did not increase. The fore-arm can now be bent to a right angle, but there is much pain in the neck, wrist, and fore-arm; the whole limb is wasted and useless, and its movements very feeble.

*Case* 6. Lambert, æt. 40, of good constitution, and sanguineous temperament, was struck on the left hip by a bale of cotton falling from a height of twenty feet. He was brought immediately to the Hôtel Dieu of Rouen, presenting all the symptoms of dislocation of the femur, the head of the bone resting a little above the great sciatic notch. After some ineffectual efforts, during which he was bled, and two grains of tartar-emetic administered, the reduction was effected. He continued well till February 11th, two days after the accident, when the thigh became swollen, and the hip painful, the patient feeling much depression. 12*th*. He spat some blood in the night—more swelling. The pulse is weak—skin yellow—hiccup, followed by some nausea—tongue covered by a yellowish-white fur. 13*th*. Vomiting of yellow matter in the night—the swelling has extended to the knee—some delirium. In the night of the 14*th* he died.

*Dissection.* A large ecchymosis beneath the skin at the anterior and outer part of the thigh; rupture of the pyriformis, gemelli, and quadratus femoris muscles. The capsule torn, as was the ligamentum teres, close to the head of the femur. In the cavity of the joint there was a quantity of reddish pus, which communicated through the rent in the capsule with a depôt of bloody pus situated between the pectineus and adductor muscles. All the great organs were sound.

*Remarks.* The death of the patient, we apprehend, was more the consequence of the accident itself, and of the constitutional irritation to which it gave rise, than of the attempts at reduction, though the latter, no doubt, had some share in producing the mischief. It is most probable, that the muscles were torn by the head of the bone, forcing its way from the socket to the sciatic notch.

As the author observes, these cases go to prove that old dislocations cannot always be reduced with the impunity which is generally imagined. He thinks, that many of the instances of paralysis of the arm, attributed to the luxation itself, are owing to the attempts at reduction, and remarks with some naiveté, "if it were otherwise, we must suppose, either that surgeons say less than they might about their mishaps, or that I alone have been unlucky enough to meet with all these unfortunate accidents." Perhaps both suppositions are partly right; at any rate, the above cases are calculated to teach gentlemen a little caution in handling their ropes and pullies.

---

### 4. EXTRACTION OF THE ASTRAGALUS.

[New York Hospital.]

A stout young lad fell from a height of 50 feet, with a hod on his shoulder, and received a compound luxation of the astragalus inwards. He was immediately conveyed to the hospital above-mentioned, and put under the care of Dr. Stevens. He lay trembling and agitated; but his skin was warm and his pulse good. The integuments and capsular ligaments over the inner side of the ankle-joint were rent, and the surface of the astragalus, where it articulates with the os calcis, occupied, and rather protruded from the wound. Dr. S. endeavoured to reduce the bone into its place; but failing in this, he tried to pull out the bone, which was also impracticable. Extension was made, and as the bone could not be reduced to its proper site, it was extracted, by dividing the ligaments that held it, with the scalpel, though not without considerable sufferings on the part of the patient. Upon minute examination of the bone, the processes to which the ligamentum inter fibulam et astragalum posterius, and ligamentum fibulæ anterius, are attached, were found broken off. The limb was laid flexed on its outer side on a splint and pillow, and the wound dressed with lint and roller. We need not detail the after-treatment, which appears to have been judicious. There was ultimately some flexibility of the ankle-joint, with little deficiency in the length of the limb.—*New York Med. and Phys. Journal.*

### 5. PNEUMONIA AND GASTRO-ENTERITIS WITHOUT PAIN.

[Val de Grace.]

The different kinds of sensibility in the viscera and in the skin have led into great delusion, not only in chronic but even in acute diseases. The following case is worthy of record on this account.

*Case.* Nicholas Lesacq, aged 52 years, a sub-officer, was received into the Val de Grace, on the 22d April, 1827, in the evening. On the morning of the 23d, Dr. Damiron, then on duty, saw the patient. His face was flushed, eyes sparkling and watery, skin burning hot, tongue dry, brown in the middle and vividly red at the sides, thirst urgent, pulse quick and cordy, breathing difficult, cough frequent, with copious ropy expectoration, dull sound in the right side of the chest, and the air not very freely permeating the other lung. The patient stated that he had been ailing since the 18th of April, with cough and fever, but that he felt no pain at any time, or in any part of the body, even on the fullest inspiration. This absence of pain embarrassed the physician a little, but an analysis of the symptoms left no doubt in his mind that there existed inflammation of the digestive and respiratory organs. Venesection—25 leeches to the epigastrium—diluents, &c. At 4 o'clock in the afternoon there came on a shiver, with stridor dentium. This rigor lasted six hours, and was succeeded by heat, but of no long duration, for the patient expired at two o'clock in the morning.

*Dissection.* Three-fourths of the right lung were hepatised, the inferior portion or fourth being sound, and separated from the hepatised structure by a very distinct line. The left lung was black, but crepitous. The bronchia on both sides were greatly inflamed, and filled with a mucous secretion mixed with frothy blood. There were no tubercles. The heart was rather larger than natural. The stomach was very voluminous, and contained only an aqueous fluid. The mucous membrane of this organ was vividly red, with several brown and white stripes, but no ulceration. Some marks of inflammation were observed in the small intestines, but not to so great an extent as in the stomach.

The foregoing case shews that several viscera may be intensely inflamed, and yet produce no local pain appreciable by the patient. Epigastric pressure caused no uneasiness—a fact which we have often observed in real inflammatory disorders of the mucous membranes, while, in many cases of simple irritation, and where no inflammation existed, we have found the greatest sensibility to pressure at the scrobiculus cordis.

### 6. PERMANENT EVIDENCE OF SUCCESSFUL VACCINATION.

[Small-pox Hospital.]

Under this head, Dr. George Gregory has published a paper in the May No. of the Medical and Physical, in which he modestly disclaims all title to much novelty—and only hopes to be useful, by refreshing the memory on certain minute points connected with vaccination. We shall give a very brief abstract of these minute points.

1. A proper vaccine scar should be distinctly defined, even after a lapse of 20 years; in order to which, it is nearly indispensable that the scab should remain on—or, at least, that cicatrisation should not be completed till the 21st day. In some cases, the cicatrix is formed by the 14th or 15th day—and then " vaccination is imperfect."

2. The true and perfect vaccine scar is circular, or nearly so. When common inflammation supervenes early, the scar is irregular in form, and the system is still open to the small-pox, more or less modified. The diameter of the circular scar is not material. The largest, however, which he considers compatible with safety, will be that of a sixpence, or small wafer.

3. The vaccine scar should be indented and radiated; though he does not insist on these appearances as a *sine qua non* in the proofs of perfect vaccination.

The sources of imperfection in the process of vaccination are chiefly the following:—effete virus; hence the inoculation should always be with fresh matter, and not by points, if possible:—pre-occupation of the system by some other important process, as dentition, visceral inflammation, fever, hooping-cough, porrigo favosa, or herpes:—and, lastly, a too advanced period of life at the time the process of vaccination is instituted.

For several collateral remarks and observations, all of a practical nature, and all indicative of the sound judgment and accurate discrimination by which the author is characterised, we must refer to the original paper in our cotemporary for May last.

In the same journal there are two papers connected with the subject of vaccination—one by Mr. Dalton, an eminent surgeon of Bury St. Edmunds, who describes an epidemic variola, against which vaccination made a bold and successful stand—the other by Dr. Morton, Physician to the Metropolitan Infirmary for Children, in which it is shewn that, in his own experience at least, the proportion of variolous attacks after vaccination does not exceed four per cent. at the most. In the cases of failure, the disease was generally very mild.

---

### 7. REMARKABLE INTERMITTENT HICCUP, ATTENDED WITH EXTRAORDINARY PHENOMENA.

[Dr. Hellis. Hotel Dieu de Rouen.]

A youth, at the age of seven years, felt a sense of fatigue rather than of pain, about the last dorsal vertebra, and soon afterwards a similar sensation in the epigastrium, which was succeeded by hiccup, at first neither severe nor of long continuance. During the space of two years, this hiccup was renewed, from time to time, and with longer or shorter intervals; but always preceded by the sensations above-mentioned, arising from the spine, and stretching to the epigastrium, without, at first, deviating from this course, or extending to other points. But, after a time, this *aura*, (to give it a name and to avoid periphrasis) began to take a wider range—darting from the epigastrium to the neck, and ultimately to every part of the body and extremities, except the head, to which it never extended. This radiation always took place immediately before and during the attacks of hiccup. In descending along the arms, this aura sometimes caused a kind of spasmodic contraction of the muscles, and clenching of the fists, during which, the hiccup would cease, for a moment, but re-appear as soon as the hand was opened. On this account, the boy often had recourse, and sometimes with success, to the expedient of clenching the fists, to relieve him from the hiccup. The attacks were, at first, separated by considerable intervals —but afterwards became almost weekly, and never ceased till the *aura* returned to the spine, the spot whence it first emanated, which generally required from one to three hours, unless interrupted by the clenching of the fists, as above-described. It is curious that the clenching of the fingers had no effect on the hiccup, before the aura descended to the hand—and whenever the fingers were relaxed, the hiccup would return. On this account, he would sometimes keep the hand firmly clenched for a fortnight together, tying a handkerchief firmly round the fist on going to sleep, lest the grasp should relax and the hiccup return. In this state he had passed two years, when he was sent to ROUEN for his education. He was there examined carefully by Dr. Hellis, in company with Messrs. Godefroy, Blanche, and Vigné, members of the Royal Academy of Medicine. He appeared to have a good constitution; his neck was short—shoulders square and expanded—appetite and sleep good—no eruptive disease had preceded the present affection —the premonitory sensation is still a sense of tension and fulness in his back. This part was examined with great care, when a circular depressed cicatrix was remarked on one side of the spine, the origin of which could not be ascertained.

For three days previous to this examination, the hiccup had been suspended by the firm flexion of the fingers of the left hand. It was ascertained, by several trials, that the least relaxation of the flexor muscles renewed the epigastric aura and hiccup, which seemed, as it were, imprisoned in the clenched fist of the patient. When the attack was renewed, in this manner, by way of experiment, the effects were sometimes alarming, the boy being threatened with suffocation, spasms, and other distressing phenomena. It was tried whether ligatures placed on the arm, or on the lower extremities, when the aura was there, would have the same effect of localising or imprisoning this electric enemy, but they totally failed, as did moxas, blisters, &c. But what was still worse, the clenching of the hand failed after a time, and a new irradiation began to dart from the spine, in various directions, exciting the hiccup, in the same way as the original aura. At length this second aura reached the fingers of the *right* hand, and these being clenched, the new enemy was imprisoned, the spasms and hiccup instantly ceasing. By way of experiment, the physicians caused the two hands to be simultaneously opened, when two distinct auræ were felt to dart from the hands in various directions over the body, exciting a most tremendous fit of hiccup! The second aura, however, disappeared in a few days, and the old enemy being once more imprisoned in the left hand, the poor boy had a truce while he kept that hand clenched. The boy was incapacitated for his studies, and sent back into the country. This state continued for a year afterwards, when the phenomena gradually ceased, and he returned to Rouen, much improved in appearance, and able to go through his scholastic exercises with ease. It is not a little curious that now a node, similar to that which we see in old arthritic subjects, occupied the index finger of the right hand, and two similar nodes two of the fingers of the left. These phenomena, we think, prove that the disease could not have been simulated, as one would be apt to suspect, had these physical changes of structure not appeared as a kind of evidence in the boy's favour.

---

### 8. DISEASED AND WOUNDED ARTERIES.

[St. Thomas's Hospital.]

In our last we noticed a paper on this subject by Mr. Travers. That gentleman having detailed some further cases in the Medical and Physical Journal for July, we shall proceed to the consideration of them here.

*Case* 1. *Aneurism in each Ham: Cure in Eight Months.* Weale, æt. 25, accustomed to carry sacks of flower, entered the hospital Nov. 14th, 1822, with a pulsating swelling in the left ham, of the size of a swan's egg, and which first appeared seven weeks previously. *Nov.* 22*d.* The femoral artery was tied with a single ligature; this came away on the nineteenth day, when the sac was much reduced in size. *Jan.* 2*d.* He was discharged in good health. In the course of a week there came on intolerable itching in the right ham, followed in a fortnight by violent cramps in the limb. In another fortnight a swelling appeared, which increased daily; and, on his re-admission, *April* 3*d*, pulsated, was very painful, and as large as a hen's egg. *April* 11*th.* The right femoral artery was tied with a single ligature, which separated on the thirteenth day. *May* 10*th.* Discharged with good use of his legs.

Mr. T. next notices a case of aneurism from the Medico-Chirurgical Transactions, in which a second operation, after the failure of the first, proved successful. He then glances at the case of a soldier, æt. 32, with aneurism in each ham, " who died on the fourth evening after the operation, of gangrene affecting the cellular membrane, which was discoloured and affected with fetid gas as high as the loin."

*Case* 2. *Diffused Aneurism, from Wound of the Artery in Bleeding.* Mary B—, æt. 20, was bled, Aug. 20th, 1820, and the wound closed as usual. Immediately a thrombus-like swelling appeared, and in the course of two days increased, so as to extend from the elbow nearly to the wrist. There was much throbbing pain and tension which was partly relieved by leeches and cold lotion. 22*d*. Copious hæmorrhage from the lancet wound, which was not stopped till syncope took place. A free incision was now made into the tumour, its contents, partly fluid and partly solid, removed, and the artery exposed. This was tied above and below the wound, which had nearly divided the vessel. 23*d*. Arm much less swollen—pulse 140—no appetite. The incision has a sloughy appearance. *Dec. cinchonæ, infus. ros.* āā ℥j. 6*tis horis. Lot. acid. sub. catap. lini.* 26*th*. The integument between the two wounds has sloughed, leaving a healthy granulating surface. From this time she continued to improve; by the 14th Sept. the wound had cicatrized, and, on the 18th, she was discharged, with an useful arm.

This case was very judiciously treated. In these diffused aneurisms, from puncture of the artery, it is generally the safest and wisest plan to secure the vessel above and below the wound. When, however, the patient applies some time after the accident, the case is different. The coagulum has become bounded by adhesions, a sac has been formed by the cellular membrane, the aneurismal tumour has become circumscribed, and now one ligature placed above it will be sufficient. This is an important distinction, both in principle and practice.

*Case* 3. *Wound of the Posterior Tibial Artery—two ligatures applied.* J. H. æt. 45, received a wound, an inch long, from a scythe, in the back of the leg, about four inches above the ankle. Profuse bleeding took place, but was stopped by a handkerchief, bound round the limb. On removing this, there was a second bleeding, which was arrested by a tourniquet on the femoral artery. He was now brought to the hospital, a distance of fourteen miles. A sponge tent was introduced to the bottom of the wound, and a roller tightly applied "from the foot to the ankle." The tourniquet was then slackened. In three hours, hæmorrhage again—tourniquet tightened. *Aug.* 21*st*, two days after his admission, tourniquet slackened—no hæmorrhage. 23*d*. Sponge removed, and adhesive straps applied. The limb is rather swollen and painful; it starts occasionally. Slight sympathetic fever. 24*th*. Hæmorrhage to twelve ounces, stopped by the tourniquet. 25*th*. An incision, four inches long was made, separating the lowermost fibres of the soleus from their insertion, and taking the direction of the wound. A cavity, containing coagulum, and fringed with lymph, was exposed, on scraping which with the knife-handle, the ends of the vessel were seen an inch and a half apart, and each was tied with a single ligature. 30*th*. Both ligatures came away—wound healthy. *Sept.* 21*st*. The patient has gone on well. A slough was thrown off from the wound in the calf. A collection has formed above the heel. *Oct.* 4*th*. Ulceration has taken place here, and has freely discharged. *Jan.* 31*st*, 1821. Discharged, cured. He has the full use of the leg.

To these cases, Mr. Travers has appended some remarks. He observes that compression by sponge, or other foreign body, cannot always be depended upon in the treatment of hæmorrhage, and that, where the ligature can be used, it is mostly preferable. "The permanent principle on which it (the sponge) acts, is that of stimulating to suppuration the fresh wound, the granulating surface of which effectually seals the bleeding orifice." With all due deference to the opinions of Mr. Travers, we conceive that suppuration is a very odd mode of plugging up an artery, and arresting hæmorrhage. We should say that the principle on which the sponge acts, is by stopping, for the time, the flow of blood, and inducing the formation of coagulum within the vessel, which is finally closed by the effusion of lymph, and adhesion of its walls. Our author, however, if we understand him aright, seems to think that the occurrence of an abscess is a security to the artery. Alluding to a case in the Medico-Chirurgical Transactions, he remarks, "the femoral artery had been divided by a carriage-wheel passing over the lower and back part of the thighs a fortnight before, and a cavity between the flexor muscles of the thigh formed, which, having gone into abscess, was for a time secure; but the ulcerative process being set up, and the wall of the abscess destroyed, the sealed mouth of the vessel was included in the destruction, and fatal hæmorrhage ensued." If Mr. T. understands, by an abscess, what is generally understood as such; to wit, a cyst, containing matter, and formed by inflammation and *ulceration*, then we contend that, so far from being a security against hæmorrhage, it rather appears to us to be the reverse. Take, for instance, the case cited above. The femoral artery was wounded, and the blood forced into the cellular membrane betweeen the flexor muscles of the thigh. Here it coagulated, and this *coagulum* was, for the time being, the security to the artery. But the effused blood became, like a foreign body, a source of irritation, and the suppurative process was set up for its removal. An abscess formed, and what was the consequence? An additional security against hæmorrhage? or a tendency to its recurrence? We should say the latter. The sponge, observes Mr. T. should not be suffered to remain in the wound longer than two, or, at the outside, three days. It should be removed as soon as suppuration occurs, and, if it be very firmly retained by adhesive matter, a poultice, applied some hours before the attempt is made, with a little patience in the operation, will always effect its removal without bleeding. If it remain in the wound for any length of time, it not only causes ulceration around it, but, by its irritation, induces neighbouring and remote abscesses in the limb. Thus, a man had the radial artery wounded; a sponge was introduced into the wound, and kept there for several days. Sloughing of the integuments of the hand, exposing the extensor tendons, followed. When this healed, an abscess formed along the upper arm and axilla, a branch of the brachial artery was opened by ulceration, and the patient sank under the bleeding.

### 9. INFLAMMATION OF THE SPINAL MARROW.

[M. Ollivier. Hôpital Necker.]

It is of great importance to accumulate facts elucidating inflammation of the spinal brain, since this is supposed by many to play an important, though unsuspected part, in many dangerous diseases.

*Case.* A young lad, aged 16 years, was admitted into the NECKER HOSPITAL, on the 3d August, 1826, ill (according to a vague and scarcely intelligible account given by himself) about three days. He seemed abstracted and confused in his ideas, could only answer in monosyllables, and was constantly moving about in his bed. It appeared, however, that he had wandering pains in his limbs, head-ache, and diarrhœa. The countenance was yellow—cheeks red, epigastrium very tender, tongue red at the sides and tip, but moist. Under the idea that the boy laboured under gastric and intestinal inflammation, he was leeched, fomented, and had diluents given for drink. Next day (4th August) the leech-bites were still bleeding, but there was no amendment. The tenderness, which was supposed to be confined to the epigastrium, was now ascertained to exist all over the body, no part of which could be touched with the finger without causing the patient to cry out, on account of pain. This excess of sensibility over the surface induced the medical attendant to suspect that he had been deceived in his diagnosis, and that the seat of the disease was in the spinal marrow. As the leech-bites were still bleeding, and the boy very weak, M. Honoré contented himself with the exhibition of diluents. The mother of the patient having come to the hospital, they learnt that he had plunged eight or ten times from the bridge of Jena into the Seine, on the 31st of August—that, at the last plunge, he had hurt his left leg, but so slightly that he walked home—and that, during the succeeding night, he had been very restless, and complained much. *5th August.* The patient was delirious, and talked incessantly, but incongruously. Mercurial frictions every six hours. *6th.* All the symptoms aggravated, except the diarrhœa. The frictions could not be properly applied, on account of the universal soreness of the body. *7th.* Extreme agitation—cries out constantly—convulsive and involuntary movements, especially of one of the lower extremities—cannot lie a moment in one position—face flushed—pulse quick and strong—skin hot. Twelve ounces of blood from the arm—small doses of laudanum—warm bath. *8th.* No alteration. *9th.* Prostration of strength—died in the evening.

*Dissection.* The membranes of the brain were sound—the cerebral substance somewhat more dense than usual. The envelopes of the spinal marrow appeared natural, and the medulla spinalis itself, at its upper part, seemed healthy. Opposite to the 7th cervical vertebra, the spinal marrow was evidently softened, and infiltrated with pus. This species of lesion extended to the 4th or 5th dorsal vertebræ, from whence, to the cauda equina, the organ was unaffected. In the chest, the lungs were sound; but the pericardium adhered to the heart throughout by a soft, cellular, lamellated tissue. There was no other organ or part in a state differing from health.

We think the inflammation of the pericardium, by which that membrane was glued to the heart, played no unimportant part in the symptoms and death of the patient.

---

### 10. HYPERTROPHY OF THE HEART; GASTRO-ENTERITIS.

[Dr. Richard. General Hospital, Evreux.]

A young man, 20 years of age, of sanguineous temperament, fell ill on the 4th October, 1823, after violent exercise in hunting. Dr. Richard found him labouring under strong fever, with sense of oppression and acute pain in the chest, particularly in the mammary regions. Twenty leeches were applied to the chest, followed by fomentations, which gave temporary relief. The irritation was next transferred to the encephalon, where a great congestion took place. The pulsations of the heart were of extraordinary violence—the head-ache intolerable—the *knees and feet swelled and painful.* Four *large bleedings* were taken from the arm, and one from the feet—sixteen leeches were applied to the neck—*pediluvia very hot—fomentations to the knees.* These measures produced no mitigation of the symptoms. Four leeches were then applied to the interior of the nares, which determined a copious epistaxis, followed by a complete remission of the symptoms. Dr. R. discontinued his visits on the 23d of October, the 19th day from the commencement of the attack.

In 1825, eighteen months after this illness, the young man entered a mercantile house in Paris, where his health was by no means good, and where he was several times bled from the arm, always with relief. In the autumn he returned to Evreux, and Dr. R. visited him in September. He was then affected with cephalalgia, dyspnœa, palpitation of the heart on walking quick or going up stairs. The pulse was full, hard, and accelerated—the appetite too great, the digestion apparently perfect. He was twice bled, and rigid regimen, with repose, was enjoined. In October, he returned to Paris, and there got worse. He was several times bled by his Parisian physician, with considerable relief. Again he returned to Evreux, and, after some imprudence in exercise, was completely laid up.

*Nov. 4th.* Pulse hard and quick—violent epigastralgia—ardent thirst—nausea—eructations, bilious vomitings—constipation—agitation—acute pains in the loins and various joints—hot and dry skin. Fifteen leeches to the epigastrium—barley water—emollient lavements. The leech-bites bled copiously for 24 hours—and the patient was greatly relieved for a time, when the symptoms returned as violent as ever, with great difficulty of breathing, pains in the chest, distressing watchfulness, palpitations, and nauseæ. Fifteen leeches were applied to the epigastrium, and produced a great discharge of blood, with mitigation of the symptoms for two days. *9th November.* All the symptoms were aggravated, and the action of the heart was frightfully tumultuous. The wretched patient could not stay in bed a moment. A large quantity of blood was taken from the arm, and the young man had a respite from his sufferings till five o'clock in the morning, when they returned with violence, and he died at nine o'clock on the 10th November.

*Dissection.* The thoracic organs were all glued together by adhesive inflammation—the pleura thickened—the lungs hepatized in several places—the heart of a most enormous size, and intimately adherent, at all points, to the pericardium, which was red and thickened. The four cavities of the heart were prodigiously dilated, especially the ventricles, the parietes of which were an inch and a half in thickness. There were large polypiform concretions in the heart, extending into the great vessels. The mucous membrane of the stomach was red, thickened, and covered with a dense coat of mucus. There were unequivocal traces of phlogosis in the duodenum and small intestines. The liver was rather large, and the other organs in their natural condition.

The reporter of the above case was called upon to open the body of a magistrate, who died in his neighbourhood, of aneurism of the heart, and there he found traces of inflammation in the mucous membrane of the stomach and intestines. From these two cases he seems very much inclined to conclude, that the abdominal phlogosis was more than an accidental complication of the cardiac malady—in fact, that it was the principal cause of the latter. We cannot, for a moment, coincide with Dr. Richard. We consider the origin of the disease to be acute rheumatism, the rheumatic irritation and inflammation (chronic or sub-acute) being ultimately transferred to the heart and membranes of the chest generally. Dr. R. remarks, that we are too apt to overlook the state of the digestive organs in affections of the heart. We would be inclined to draw the opposite conclusion, and say, that we are too apt to look upon real organic disease of the heart and lungs as merely sympathetic affections, resulting from disorder of the digestive organs.

The foregoing case affords another instance of the bad effects of large and repeated bleedings in acute rheumatism. Every day's experience furnishes us with proofs of the danger of metastasis from such violent depletion in this peculiar disease. The metastasis may not be immediate —but it may be more dangerous, because more insidious, in the slow form of movement which it pursues.

---

### 11. ON PULMONARY APOPLEXY.—DR. BOUILLAUD.

[Hospital Cochin.]

The above term sounds rather oddly to English ears; but it is by no means an improper term, and the authority of Laennec is a sufficient passport for its introduction into the pathological nomenclature of the present day. Laennec was the first who traced, with any degree of minuteness, the nature of pulmonary apoplexy, or hæmorrhage; but many important additions have since been made to our knowledge of this serious malady. It is with the view of filling up lacunæ, that the distinguished Physician of the Cochin Hospital comes forward on the present occasion.

*Case* 1. E. T. Gerault, aged 58 years, Professor of Belles-Lettres, of sanguineous and strong constitution, with large chest, and otherwise well-formed, was admitted into the Cochin Hospital on the 22d September, 1822. He stated that he had been ill for six years, after a voyage to a tropical climate. Since that voyage, he had been subject to what he called attacks of *asthma*, especially in cold and damp weather. The intervals were of various duration, but they had become shorter, and the attacks more severe, since a suppression of the hæmorrhoidal discharge. M. Gerault had been at the hospital two years previously, for the same complaint, and was considered to be labouring under aneurism of the heart. In 1821, he had spat up some blood for a fortnight. Some time subsequently, after a long walk, he became affected with dyspnœa to a great degree, so that he could scarcely move without a sense of suffocation, constriction in the centre of the thorax, and decubitus difficilis on the left side, some cough, demi-extinction of voice, regular strong pulse slightly accelerated, tongue white and moist, trifling diarrhœa.

At 11 o'clock at night, of the first day in the hospital, Dr. B. was summoned, and found the patient in the following condition:—sitting up in bed, breathing with difficulty—face pale—eyes haggard and prominent—cold perspiration over face and body—continually rocking from side to side—great wheezing in the bronchia—all the respiratory muscles in strong action. The pulsation of the heart could not be heard or felt—the *former*, probably from the great wheezing, the *latter*, from the violent agitation of the chest in respiration. The pulse was precipitate and irregular, the voice almost extinct, the patient earnestly supplicating for relief. M. B. bled him on the spot. The blood was black and thick. It issued with difficulty, at first, but afterwards sprang out with force. Sixteen ounces were abstracted. This measure, together with a sinapism to the back, and some antispasmodic medicine, produced a degree of relief, and the patient was able to lie down, with the head well elevated. 12*th September.* The patient felt himself much better; but still his condition appeared very alarming to his physician. The mucous wheeze (râle muqueux) continued—the sputa were viscid, and tinged with blood—breathing very short—face of a violet colour—lips livid—pulsation of the heart imperceptible by the hand—audible faintly in the front of the sternum—pulse soft and embarrassed. Cupped and scarified on the chest—colchicum and expectorants. 13*th.* The amelioration was considerable this morning; but the face was red and partially livid. 14*th* to the 30*th.* The respiration is sometimes free, sometimes impeded—countenance pale—hands blue. During the first eight days of October, the patient spat up a large quantity of black blood—the legs swelled. On the 13*th October*, there was another abundant hæmoptysis, and on the 14*th*, he died.

*Dissection.* The right pleura costalis and p. pulmonalis were adherent—in the left pleura, there was a pint of reddish fluid. Both lungs presented the cadaveric engorgement at their inferior parts (which happened to be the *anterior*, as the body was turned on the face soon after death)—the posterior parts of the lungs were crepitant. A considerable proportion of lung, however, was found in a state very different from that of mere cadaveric congestion—namely, with a quantity of blood extravasated in the air-cells, and combined, as it were, with the pulmonary structure itself, forming a substance resembling spleen. There was, therefore, but a small proportion of lung which was permeable to the air. The bronchia were gorged with sanguinolent mucosities, and the mucous membrane was of a violet colour. In the pericardium, there were a few ounces of fluid, of a sanguinolent and flocculent character. The heart, which was gorged with blood, was double its natural size, and, together with the effusion, encroached greatly on the lung of that side. The auricles were dilated in their cavities, and thickened in their parietes. The walls of the ventricles, especially the left, were in a state of hypertrophy. There were several other alterations of structure about the heart and arch of the aorta, which we need not specify.

In the abdomen, the liver was found gorged with blood, and, in fact, in nearly the same state as the lungs. The mucous membrane of the stomach was injected, and, in some places, inflamed.

The other case is not materially different from the one we have detailed, and may be passed over. It is this state of pulmonary engorgement, or apoplexy, which generally gives rise to hæmoptysis, the latter not resulting, ordinarily, from the rupture of any large vessel. In ulcerations, however, of the lungs, as in ulcerations of the intestines, the blood may and does come from the mouths of open vessels, and not by a species of exhalation.

The ausculatic characters of pulmonary apoplexy, as laid down by Laennec, are the absence of respiratory sound in the part, and the râle muqueux. But it is not very easy always to distinguish this engorgement from common hepatization, and other morbid states, in which the cells of the lungs are not permeable by the air.—*Archives Generales.*

---

### 12. CASE OF ACUTE SCURVY. PURPURA HÆMORRHAGICA.

[Hôpital St. Louis.]

J. Delsalles, aged 27 years, of strong constitution, was in the habit of sleeping in a damp and ill-ventilated apartment, which so affected his breathing that, in hot weather, he usually kept the window of the apartment open. Having one day committed an excess in drink, he felt unwell that evening; and, in the course of the succeeding day, he perceived petechiæ spreading over his body. Not being ill in health, Delsalles paid no attention to these petechiæ, at the time; but the spots gradually increased, in number and in size, and at the end of six weeks, a violent epistaxis came on, which could not be checked by any of the means used. It was on the 5th day of this epistaxis, that the patient entered the Hôpital Saint Louis, viz. 5th July, presenting the following symptoms:—extremely numerous ecchymoses dispersed over the whole surface of the body, varying in size, from a millet seed to that of twenty sous piece, and some even larger. Some of them resembled flea-bites

—they were not elevated above the surface of the skin—their colour was between a pale red and a purple. Blood, very fluid, but rather pale, dripped slowly from the nose and gums—the tonsils, palate, and fauces appeared of a blue colour—face pale. Yet the patient did not appear to be much weakened, and he had walked that day twice to the hospital, before he was admitted into it. M. Richerand pronounced the disease " un scorbut três grave," and ordered antiscorbutic ptisans, with wine, &c. Towards the middle of the day the patient began to complain of head-ache, which soon became violent, the pulse being small and concentrated. Cold to the head, sinapisms to the feet. At midnight, the epistaxis, which had ceased from midday before, re-commenced, and blood came from the ears—breathing very difficult—and at each respiratory movement, a quantity of frothy blood came from the mouth. The ecchymoses now developed themselves in the conjunctiva. The pulse remained in the same state. At three o'clock next morning, the patient lost the use of his intellectual faculties and of his voluntary muscles, except those of one arm. The breathing became laborious—the discharge of blood from the mouth very copious, so that the patient's bed was inundated. At 5 o'clock *he was bled to eight ounces*, and in a few minutes expired.

*Dissection.* The vessels of the pia mater were distended with black blood; and this membrane presented also a large infiltration of blood towards the middle and superior portion of the right hemisphere. This hemisphere shewed at its interior face, near the posterior extremity of the corpus callosum, a fluctuating tumour, from which there issued a quantity of blood, when punctured. Blood was found in the lateral and middle ventricles. The cellular tissue surrounding the dura mater of the spinal canal was infiltrated. There were ecchymoses on the pericardium and heart, and some sanguinolent serosity in the bag of the pericardium. The heart itself was pale and flaccid—the ventricles empty—ecchymoses on the surface of the carneæ columnæ—aorta, pulmonary artery, and vena cava of a red colour internally, and contained fluid blood. There were some ecchymoses on the external and internal surfaces of the aorta. In the larynx there were several ecchymoses—trachea very red, and, together with the bronchia, presenting numerous ecchymoses—much blood in the mucous follicles—many points of extravasation in the parenchyma of the lungs—much sanguinolent serum in the cavities of the pleura. The tonsils were of a violet colour, œsophagus contracted and pale—internal surface of the stomach of a purple tint—black fluid in the stomach and duodenum—various ecchymoses in the course of the small intestines, and on the peritoneum—spleen almost exsanguious—liver sound—skin covered, in various places, with ecchymoses.—*Bibliotheque Medicale.*

If the above case does not shew that there is some truth in the " humoral pathology," we are much mistaken. At the time we translated this case, we were in attendance on an interesting one of a similar character, with Mr. Pretty, of Mabledon place, some particulars of which may not be uninteresting. We shall give them from Mr. Pretty's notes.

"*Case*. G. Oldney, a carpenter, of temperate habits, became affected with subacute inflammation of the mucous membrane of the larynx and trachea in January 1827. By proper measures these symptoms were mitigated, and he resumed his work. But again they returned, and with them a discharge of blood from the mouth and throat of a dark purple colour. On inspecting the mouth, some spots were seen on the inside of the cheeks, elevated above the surrounding surface, and discharging blood. Some of them were the size of a sixpence, and elevated the thickness of a half-crown piece. These spots ultimately affected the velum pendulum palati. Blood was also brought up by hauking from the fauces, and probably from the air-passages. The pulse got up to 120, and even 140, without strength or fever. On the second or third day, petechiæ began to appear on the legs. Dr. Johnson visited the patient with Mr. Pretty, and small doses of ol. terebinth. were prescribed, with compound tincture of bark, and astringent gargles. Next day the pulse fell to 100. He continued to lose blood, but in less quantity. The turpentine was increased, and the sulphate of quinine was ordered. The blood continued to ooze from the mouth and throat for many days, but not to any great extent. Some also appeared in the urine. The petechiæ ultimately went off, and the hæmorrhage ceased. But now the symptoms of laryngeal disease advanced. He went off to Devonshire—had a dreadful passage by sea—and ultimately died of laryngeal phthisis.

### 13. REMARKABLE TENDENCY TO PHLEGMASIÆ.

[M. Lomel. Military Hospital, Strasburg.]

This case is recorded in M. Broussais' "*Annales de la Medecine Physiologique*," a journal which we have only recently procured in exchange, but which will hereafter be more known to our readers. There is a passage in the preliminary discourse to the first number for this year, which we shall extract, as not a little applicable to our own times and country. M. Broussais is speaking of a new French quarterly journal, on a plan nearly similar to our own, and which is to be conducted without any party-spirit, but solely for the purpose of collecting the rays of knowledge from all quarters, foreign and domestic—and then diffusing them in a concentrated form, through the various ramifications of medical society.

"Nous ne pouvons qu'applaudir à des vues aussi sages, aussi philanthropiques. Il était temps que les gens de bien se réunissent pour neutraliser les efforts de *ces coteries* où l'on excite *les passions* contre les hommes de la science qui n'en font pas partie."

We sincerely hope—indeed we are thoroughly convinced that the tide of dissention and party-spirit which has, of late, unhappily overflowed, in this country, is now rapidly ebbing—an event that must give great satisfaction to all those who wish well to medical science, and desire the respectability of its professors.

*Case.* A sub-officer of artillery, aged 23 years, of sanguineous temperament, entered the Military Hospital of Strasburg, on the 5th of December, complaining of acute pain, of four days' duration, in the lower part of the left side of the chest, verging round towards the epigastrium. The patient affirmed that he felt this pain first in the spinal column, and that it afterwards extended forward. Deep inspiration caused much inconvenience, as well as coughing, which last, however, rarely occurred. The left ribs were nearly motionless in the act of breathing—percussion there caused pain, but elicited a clear sound. There was but little increase of heat—the appetite was gone—the tongue white in the middle, and rather red at the tip and sides—bad taste in the mouth, which was dry and clammy—thirst—pulse frequent and hard. He slept pretty well, and the bowels were regular. Twenty leeches were applied to the affected side—he was put on the lowest diet —and diluent drinks were prescribed.

6th. The leeches bled copiously, and the pain was much diminished; but the skin was dry and hot—the pulse quick, but less hard—thirst, confined bowels. *Emollient lavement.* *7th.* Same state—same prescription. *8th.* The bowels opened; but the symptoms nearly the same. Fifteen leeches to the anus. *9th.* Passed a good night—pain of side gone—can breathe freely. Pulse is still quick and hard.

10*th.* Acute pain in the right thigh, where a phlegmonous swelling appears. Thirty leeches to the thigh. 11*th.* The tumour diminished. Some febrile symptoms still remain. 12*th.* Another inflammatory swelling has appeared in one of the arms. Twenty leeches to this part. 13*th.* The swelling on the arm is diminished; but a similar phlegmon has appeared on the corresponding knee, to which 20 leeches have been applied. 15*th.* Inflammation has occurred in the ankle of the same extremity. Ten leeches to this new phlogosis. 18*th.* Some pain of the head is complained of, the other local inflammations having much subsided. 20*th.* To the head-ache were added swelling of the left side of the face, and erysipelas. The constitutional disturbance is now considerable. 21*st.* The swelling of the face is so great that the patient can scarcely open his eyes. A pustular eruption is appearing over the body, as large as variola. The febrile symptoms aggravated. 22*d.* The patient is delirious. Twelve leeches were applied to the temples; but the appearance of the erysipelas and the constitutional symptoms leave little hope of life. He died next day.

*Dissection.* Under the seat of erysipelas in the face, there was a depôt of purulent matter, and denudation of some of the bones. There was purulent matter, of no good quality, found in all the other seats of inflammation, above alluded to. There were six ounces of purulent matter found in one of the knee-joints, the synovial membrane being much inflamed. The vessels of the pia mater were highly injected, and some portions of the brain were positively inflamed—the cerebral substance being generally firmer than natural. The vertebral canal presented a large quantity of bloody fluid infiltrated into the cellular tissue, but the medulla spinalis itself was firmer than natural.

In the chest there was very little variation from a state of health, except some pleural adhesion. In the stomach several zones of vascularity were observed in the mucous membrane, together with thickening and softening of that tissue. The internal surface of the small intestines was red throughout; some patches of inflammation in the large intestines. There was no other disease in the abdomen.—*Annales de la Medecine, &c.*

The above case is certainly remarkable for the powerful tendency to inflammatory action in different tissues, and consequently presenting different trains of phenomena. M. Broussais justly remarks that such inflammatory tendencies can only be successfully treated by early and energetic depletion. Had such measures been employed in the above case, it is probable that the inflammatory irritation would not have spread from one tissue to another, with ultimate destruction of life.

### 14. STRANGULATED HERNIA.*

[St. Bartholomew's.]

The more we see and read of strangulated hernia, the more we are convinced of the advantages and success of an early operation. We are quite sure that five out of ten of those who have died after operation, would, in all probability, have been saved by an earlier use of the knife. Mr. Earle has related two cases which bear upon the question.

*Case.* J. E. æt. 30, was admitted into hospital, April 27th, with a large scrotal hernia. This first took place at the age of twelve, in consequence of a blow; it was reduced, and he applied a truss, which, however, he left off for ten years, during which time the gut only descended occasionally, although he exerted himself much as a drayman. For the last four years the hernia had been more troublesome, and obliged him to return to his truss. Notwithstanding this was on, the gut descended at half-past nine on the morning of his admission, and could not be reduced. It rapidly increased in size, and became tense and painful. At half-past twelve Mr. Earle saw the patient. He was placed in a warm bath, bled to faintness and the taxis employed, without effect. A tobacco glyster was then thrown up; it produced depression of the pulse and sickness, and the taxis was tried again, but the tenderness of the tumour and abdomen increased so as to call for an immediate operation. This was performed at two P. M. The tumour distended the scrotum, and the testicle could be felt at the back of it. In the sac there was much fluid, and a fold of small intestine, seven inches in length, much discoloured, and closely girt at the external ring, the stricture of which was increased by the protrusion of a portion of thickened and congested omentum. Although both the sac and ring were fairly divided by the knife no impression could be made either upon the gut or omentum. The inguinal canal was laid open, and some

* Med. and Phys. Journ. July.

healthy intestine drawn down, but the stricture at the inner ring, was so narrow as to require " a *third* application of the hernial knife."* The omentum was now returned, and after drawing down some more intestine the reduction of the whole was after a time effected. The testis was seen at the bottom of the sac, inverted with the tunica albuginea. The edges of the wound were brought together with sutures, &c. as usual, and a purgative enema thrown up with magnes. sulph. ℥ss. every three hours. At 5 P. M. there was pain in the abdomen, which was relieved by the abstraction of twenty-four ounces of blood. Not a bad symptom followed and the wound healed by the first intention.

*Remarks.* Mr. Earle's practice was decided, and, in our humble opinion, highly judicious. Another surgeon might perhaps have temporised—might have said—" Let us wait and see what a little rest, and the taxis afterwards, when the parts are quiet, will do for the patient." Why, ten to one that it would have done that, which no subsequent operation could have undone,—either induced mortification in the gut, or given rise to a fatal peritoneal inflammation. The rupture, in this case, took place at half past nine, the operation was performed at two, and yet in this short space of five hours, the stricture was rendered so complete, that it was necessary to divide the external ring, inguinal canal, neck of the sac, and inner ring, before it could be relieved.

*Case* 2. C. M. æt. 29, was admitted at midnight, May 1st, with strangulated hernia. When eight years old the right testicle descended, and soon after he discovered a rupture on that side; for this he tried to wear a truss, but it caused so much pain in the testis that he left it off. The rupture could always be reduced till the preceding night, when the tumour felt tense. In the morning it was larger, and he vomited every thing. A medical man now prescribed purgatives, but these giving no relief, he was sent to the hospital.

Mr. E. saw him at two A. M. The tumour was tense, and very painful, there was tenderness in the lower part of the abdomen; the pulse was feeble; countenance anxious; and skin clammy. The operation was resorted to forthwith. On cutting through the integuments, the sac was found to be narrow for an inch and a half from the ring, and then to swell out into a large globular tumour. On puncturing this, some fetid fluid was discharged, and a large fold of small intestine exposed; its surface was rough and highly discoloured. It was girt by a firm annular contraction at the lower part of the tunica vaginalis of the cord; " the sac being formed by that portion which should have enveloped the testicle, and which had assumed the size and shape of a large hydrocele." The vaginal canal was slit up, and the opening found to communicate directly with the abdomen, there being, in fact,

* " I mention this circumstance, the rather as in a publication (the Lancet) which professes to give correct hospital reports, it is stated that the hernial knife was not used at all !!"

no distinction between the outer and inner ring. The gut immediately above the stricture was comparatively sound, some of it, therefore, was drawn down, and the contents of the diseased portion gently pressed into it. Mr. E. hesitated whether he should attempt to return this, but a slight pulsation appearing in the arteries of the part, he divided the neck of the sac at the ring, and cautiously reduced the intestine. 3 A. M. Pain in the abdomen; v. s. *ad* ℥xxx; two enemata, which brought away four stools. At 8 A. M. sixteen ounces more blood were taken away, and a saline draught with magnes. sulph. ʒj administered. At night, he took a dose of calomel and opium, followed by castor oil, next morning. From this time he went on favourably, under a water-gruel diet, and occasional application of leeches. On the 27th, it was necessary to bleed him for pain in the seat of the disease, and pain in the abdomen. On the 27th, fluctuation being felt above the wound, it was punctured and six ounces of good pus discharged. In a week the abscess closed, and he is now walking about the wards convalescent.

*Remarks.* Here is another instance of the rapidity with which strangulation, or something near akin to it, succeeds the descent of the intestine. In 24 hours the disease had made such progress that Mr E. observes "In the course of my experience, I never met with an instance of recovery where the intestine was so much discoloured." Had the case not been treated with the promptitude and decision with which it was—had the operation been delayed but for one short hour, there is every reason to believe that irremediable disorganization would have taken place, and the patient's fate have been irrevocably sealed. Mr. Earle remarks that the situation of the stricture is by no means common. Wrisberg gives a similar case, and so does Mr. Lawrence. We quite agree with our ingenious author, that the abstinence from food and medicine, with the depletion, general and local, mainly contributed to the fortunate result.

---

### 15. COLICA PICTONUM.*

[Hôtel Dieu d'Orleans.]

It appears that, in and about Orleans, there are great numbers of people employed in white-lead manufactures, and consequently the author of the volume under review, as chief physician of the Hôtel Dieu of that city, has had great experience in this painful and sometimes dangerous disease. Between the years 1820 and 1826, he has treated 147 cases of colica pictonum in the above hospital, without the loss of a single patient. The statement is perfectly authentic, as he has given the name of every individual, with the date of his entry and discharge, so that any misrepresentation would be immediately detected by the medical officers of the hospital.

---

* Memoire sur les Empoisonnemens par Emanations Saturnines. Par M. Ranque, Medecin en Chef de l'Hôtel Dieu d'Orleans. 8vo. 1827.

The following are the symptoms which this disease has generally presented to Dr. Ranque:—At the beginning, distaste for food—nausea—slight salivation—disagreeable dreams:—soon afterwards, there is vomiting of the alimentary matters mixed with bile of various colours, yellow, green, black, &c. together with some mucosities. Then we have severe pains about the umbilicus, in the hypochondria, in the epigastrium, loins, and iliac fossa, returning at intervals—these intervals being long at the beginning, but gradually shortened as the disease advances. The pains are always worse at night than in the day. They are generally accompanied by head-ache, pains in the knees, calves of the legs, ankles, soles of the feet, and insides of the thighs. These pains frequently alternate with those of the abdomen. They seldom attack the wrists, elbows, or shoulders. The abdomen is generally insensible to pressure, without any change in its natural temperature. There is no increased action of the arteries in any part of the body—no redness or dryness of the tongue—no thirst—no pyrexia—pulse generally below 60 in the minute. The constipation is most obstinate, and when stools are procured, the fæces are in little balls like goat's dung. The urine is usually clear and abundant. The expression of the eyes and countenance is altered, and indicative of suffering and inquietude—the *morale* of the individual is in a state of complete prostration. In a few cases, comparatively speaking, there were symptoms of inflammation in the abdomen. In a still smaller proportion of cases, the disease assumed a terrific form, being complicated with cerebro-spinal affection, the marks of severe organic changes being found in the brain, spine, and organs of sense.

The author does not propose his mode of treatment for those cases in which there are symptoms of inflammation in the abdomen. This inflammation he considers as quite a supervention, and in no way essentially connected with true colica pictonum, which is an affection of the nervous system quite independent of phlogosis. When abdominal inflammatory symptoms appear, the disease is to be treated by leechings alone, and the treatment of the colica pictonum is to be, for the time, suspended.

In the uncomplicated cases of colica pictonum, characterised by the symptoms already detailed, our author has invariably succeeded by the *methodus medendi* now to be described. This method consists in a plaster over the abdomen—another over the loins—glysters—and a certain potion internally. The plaster for the abdomen is composed of an ounce and a half of emplastrum galbani compos:—the same of emplastrum lyttæ—half an ounce of " thereaque"—one drachm of camphor, and half a drachm of sulphur. These are to be incorporated, secundum artem, and spread on a large piece of leather which may cover the whole front of the abdomen. Previously to its application, however, the plaster is to be warmed, and a drachm and a half of tartar emetic, mixed with some camphor and sulphur, is to be spread over its surface. The plaster over the loins is to be made in the same way as that for the abdomen, with the exception of the tartar emetic, which is to be omitted. Those parts which are in pain, in any other part of the body, are to be rubbed with a liniment, into the composition of which, the extract of belladonna enters pretty freely. A mixture is also taken internally, each dose of which contains 20 drops of the etherial tincture of belladonna, (one ounce of the powdered leaves infused for three days in three ounces of sulphuric ether,) drinking plentifully of barley water, with or without gum arabic. The patient is to take no solid food whatever, and indeed as little of any kind of nourishment as possible. The lavement made use of by Dr. Ranque is composed of decoction of linseed—four ounces of oil—and twenty drops of the tincture of belladonna above-described. The lavement and the potion are to be taken every day. On the third day, the abdominal plaster is removed—the lumbar one is allowed to remain on. If, however, the colicky pains should not have ceased by the third day, which they generally do, the abdominal plaster is to continue on some time longer. In such cases there will be a plentiful crop of antimonial pustules, and much cutaneous irritation, for which fomentations and poultices must be employed.

The cure required from two to twenty-five days. One hundred and twenty-nine were cured between the second and twelfth day—the remainder between the thirteenth and twenty-fifth day. In almost every case, the vomitings ceased on the second day of the treatment. By the fifth day, the abdominal pains ceased in the great majority of cases. On the sixth day the pains in the limbs ceased, and the constipation was overcome soon afterwards, which was the prelude to restoration of health.

The success which Dr. Ranque obtained by this method, in colica pictonum, unaccompanied by any fever or inflammation, induced him to extend it to some other affections, viz. to chronic vomitings, (of apyrectic character)—to tetanus, (not traumatic)—to epilepsy, considered dependent on abdominal derangement—to puerperal mania, &c.—and in various cases he has been fortunate.

From what appears in this volume, it is evident that the author, although he lost none out of the 147 cases recorded in the tables, lost other cases which are not detailed. He says, indeed, that these were of a different character—that they were complications—and that the patients died of the supervening affections. But still these cases ought, in all fairness, to have been given. Assertion is no proof in medical matters. We agree with the author, however, in believing that colica pictonum is originally and commonly an affection of the nervous apparatus of the alimentary canal; and that it affects other parts of the system consecutively. We believe that the disease may run its course, without materially or evidently affecting the vascular system. But there are cases in which both systems are affected, and then a modification of treatment is necessary. It is evident that the principle of Dr. Ranque's treatment is founded on counter-irritation externally, and sedatives internally—the laxative injections being no more than is indicated in all torpid states of the bowels. The counter-irritation, so powerfully effected by the extensive application of the antimony, must doubtless make a great and salutary impression on the disease, and it is worth the attention of practitioners, when they meet with this painful malady.

We shall here introduce a case and some observations by Dr. Pascal on the COLICK OF MADRID, a disease nearly allied to, if not identical with, that under consideration.

*Case.* H. Henry, a soldier of the first regiment of infantry, aged 22 years, of good constitution and bilio-sanguine temperament, entered the Military Hospital of Madrid on the 9th May, 1824, for an intermittent fever, which disease persisted till the beginning of July. At this time, he was seized, without any apparent cause, with obscure deep-seated pain in the abdomen, obstinate constipation, and dysentery, without either dryness or redness of the tongue. The abdominal pains soon became excessive, and to these were added pains along the spine, and under the sternum, as also along the arms. From this time the patient had no rest—the insomnium was constant—eyes haggard—the muscular movements irregular—the temper irritable—great prostration of strength. Paralysis of the upper and lower extremities now appeared—and the bowels, which hitherto had remained obstinately confined, became soluble. He died on the 17th July. Diluents, antiphlogistics, and warm baths, were the only means employed.

On dissection, the membranes of the brain were found highly injected; but the cerebrum and cerebellum were in their natural condition. There was some serous fluid in the lateral ventricles—the spinal marrow was highly injected, and firmer than natural—the lungs were gorged with blood, but shewed no trace of inflammation. The tissues of the heart were much injected, and the internal surfaces of its chambers were red. The mucous membrane of the stomach was pale—the intestines natural. The ganglia of the abdomen were almost all red, and most of them apparently enlarged.—*Mem. de Med. et Chirurg. Milit. Vol.* xix. 1826.

In the same volume Dr. Pascal relates several other cases of the Madrid colic, in which the ganglionic system presented similar appearances. Hence he concludes that the traces of inflammation which are found in these subjects, are consecutive of the original affection of the ganglionic nerves.

In a memoir published in *the Journal Complementaire*, by Dr. Marquand, the same view of the Madrid colic is taken. This gentleman had the charge of the Military Hospital of ST. JACQUES DE COMPORTILLE, in which institution there were never fewer than 40 or 50 patients affected with this disease. The following is the course which the malady generally pursued, according to his observation;—slight and transient pains were first complained of, in the line of the colon, especially of its transverse arch, without any material disturbance of the functions. Afterwards moroseness of temper was conspicuous—inappetence—difficult defecation, but not decided constipation—much flatulence and discharge of wind. This state lasted two or three days, at the expiration of which, all inclination to stool ceased, and the flatulence disappeared. From this period, gastric symptoms were developed. There was pain in the epigastrium—pallid and dejected countenance—small, slow, and contracted pulse—scanty urine—dry but not hot skin. The patient was inclined to sit with his arms folded on, and pretty forcibly compressing the abdomen, the trunk of the body bent forwards. To these symptoms succeeded hiccup and vomiting of glairy and bilious matters. No sleep. The belly now became flattened, and pain was complained of in the right hypochondrium, and sometimes in the umbilical region, but without any diminution of the epigastric pain. The eye became yellow, and the yellowness spread to all other parts of the body. These symptoms gradually increased, and led to the tomb:—sometimes with mere marasmus—sometimes with paralysis. In the course of the disease there were occasional remissions which promised convalescence, but these were deceptive.

The treatment which proved most successful in this terrible disease was a combination of opiates and purgatives.

### 16. UNUNITED FRACTURE CURED BY PRESSURE.*

[St. George's Hospital ]

In a note to the article on "carotid aneurism" in our last number, we alluded to this case, but gave no particulars of the treatment. These have since been detailed by Mr. Brodie in our esteemed contemporary for July, and it may not be uninteresting, just to glance at the means by which the cure was effected.

J. M'Ewen, æt. 24, broke his right arm and left leg, in November, 1825. He was taken to a hospital and treated in the usual way. The fracture of the leg did well, but no union took place in the arm. In August, 1826, he went to Panton Square, when Mr. Wardrop passed a seton, which was withdrawn at the end of a week. After a time the patient was discharged, and reported as *cured* in the Lancet. In Nov. 1826, he entered St. George's, the broken ends of the bone appearing to be united by ligament, riding one over the other, and admitting of extensive motion. Mr. Brodie now determined on applying pressure, on the principle suggested by Mr. Amesbury. The fore-arm being semi-bent, a wooden splint adapted to its figure, and reaching from the axilla to the fingers, was applied on the inside. On the outside of the arm, a straight splint was placed, extending from the shoulder to the outer condyle, and both splints were then secured by bandages. Over all there was a tourniquet, the band of which embraced the fracture, whilst the degree of pressure thus made on the broken bone was easily regulated by the screw, which was on the outside of the arm. The splint on the inside being broader than the limb, and only slightly concave, the principal vessels were defended from pressure, and whatever was the force employed, the circulation was but little interrupted. In six weeks, the motion of the fractured bones was much diminished, and

* Med. and Phys. Journal.

at the end of three months none was perceptible. On the 31st May, the man left the hospital, the bones being firmly consolidated, and the arm as useful as before the accident.

This case does credit to the ingenuity and perseverance of Mr. Brodie, and we have no doubt that pressure, properly and steadily applied, will prove effectual in many, if not most, of those cases of ununited fracture, which have been not unfrequently an opprobrium to surgeons and surgery.

### 17. CUTANEOUS DISEASES.

[Meath Hospital—Dublin Infirmary.]

The following cases are recorded by Drs. Graves and Stokes, from their clinical practice in the above-mentioned institutions.

*Case* 1.—*Psoriasis.* A stone cutter, aged 40, was admitted with scaly eruption covering the back and right shoulder—the skin beneath the scales being of a bright red colour. The pulse and functions were natural. Several venesections were employed, together with repeated applications of leeches and warm ablutions. Purgatives were also freely administered, and saturnine lotions applied. Under this plan of treatment he was almost cured; but had a partial relapse, probably from irregularity of diet. The decoct. sarsæ was then given, with supertartrate of potash and tincture of squills. He was soon dismissed cured, having used the unguent. hyd. nitrat. occasionally, mixed with tar ointment.

*Remarks.* The utility of the decoct. sarsæ with the medicines above-mentioned, in chronic diseases of the skin, was learnt from Professor Autenriette, of Tubingen. This remedy has been found useful by the authors, in prurigo senilis—a disease frequently accompanied by defective secretion of urine.

*Case* 2. *Porrigo.* J. Conolly, aged 40, had been several weeks in hospital for chronic rheumatism, when the scalp became affected with redness and soreness, succeeded by a pustular eruption exuding a copious purulent secretion which concreted into scales. The hair was shaved—the scalp poulticed and leeched several times, with marked benefit. The inflammation and eruption having abated, a saturnine lotion was applied, and ultimately an ointment composed of equal parts of the unguentum hyd. nitrat. and tar ointment. The cure was soon completed by this plan.

*Remarks.* The utility of leeching the scalp, in this recent case, was very evident—and the authors have derived much benefit from the same means, even in chronic cases.

*Case* 3. *Peculiar Swelling of the Hands.* This disease, very common in Ireland, was first described by Dr. Graves in the 4th volume of the

Dublin Hospital Reports. The following case is offered as an example, not only of the disease but of the best method of treatment. Catherine Smith, aged 15, became affected, four years ago, with swelling and redness of the right hand. It declined in a few weeks, and then regularly returned every month afterwards. Between the attacks, the hand still remained swollen, and pitted a little on pressure. Two years afterwards, the left hand presented the same phenomena. When received into hospital, both hands were much swollen, particularly the backs and fingers. The monthly exacerbations of the complaint still continue, the hands becoming very red at those times. Her eruption had come out about the wrists a few weeks previous to admission, and had dried into scabs. The general health was good, and the functions undisturbed. Leeches and poultices were twice repeated, and then cold lotions applied. After this she was bled from the arm, and had the sulphate of quinine internally. By these means she was cured.

*Remarks.* When tolerably recent, or when the swelling is not very great, this disease may be cured by a *perseverance* in the *antiphlogistic* plan during the exacerbations; and by the application of moderately light bandages, and the internal use of sulphate of quinine or arsenic, during the intervals.

*Case* 4. *Inveterate Psoriasis.* Eliza Congreve, aged 12 years, was admitted on the 25th October, universally affected with scaly desquamations of the cuticle, the skin being of a bright red colour beneath the scales, which were of a silvery appearance. The pulse was 100, and strong, with thirst. Two venesections were performed, and brisk purgatives were given, then the tepid bath. Under this treatment the patient improved considerably—the redness of the skin faded—the scales less numerous—the itching entirely gone. The perspiration of the skin was quite defective since the commencement of the complaint. The decoctum sarsæ cum supert. potassæ was given twice a day, and the tepid bath every night. Mercurial aperients were also given. It required a considerable perseverance in these means, aided by sulphur baths, and sulphur internally, together with alkalis, leechings, bleedings, and the ointment already described, before the patient was cured.

*Remarks.* This inveterate psoriasis yielded very satisfactorily to the remedies employed. "When cutaneous inflammation has been sufficiently subdued, the internal use of sulphur often proves of great utility in altering the action of the skin." A slight increase of excitement sometimes follows its use; but this soon subsides, especially if the antiphlogistic measures have been carried to a proper extent previously.—*Clinical Reports, &c. By Dr. Graves and Dr. Stokes, Dublin,* 1827.

The physicians above-mentioned deserve great credit for the zeal and intelligence with which they have recorded their practice in the institution to which they belong. They form a striking contrast to the conduct of many of the drones who batten on the public in this country! If this last class escape our scrutiny, then there are no snakes in Virginia.

### 18. FEVER—GASTRO-ENTERITE.

[Bartholomew's.]

Instances like the following will tend to open the eyes of English practitioners to the state of the stomach and bowels in fever, and to the effects of repeated purgation in this disease. Our readers are aware how often we have endeavoured to draw their attention to this important subject. But the silly prejudices of John Bull against any thing of French origin, have greatly contributed to the perpetuation of error.

*Case.* An Irishman had been attacked with fever, and treated in the Fever-Hospital, under Dr. Tweedie, who ultimately sent the patient into St. Bartholomew's Hospital, "from the supposition that syphilis had supervened; a bubo having manifested itself in the groin, and a putrid sort of ulcer being discovered on the penis." When admitted, the reporter remarked a wildness of expression and flushed state of the countenance, general tremor of the muscles, and mental imbecility, "sufficiently characteristic of the state and stage of existing fever." He had a dark brown tongue, somewhat moist, and a soft compressible pulse at 100 in the minute—depression of vital powers. In the groin was a large circular slough, emitting a putrid odour. A similar slough was seen on the dorsum of the penis. Mr. Lawrence, supposing that the patient was in the last stage of fever, merely exhibited a few grains of rhubarb, and ordered cold applications to the head, with poultices to the ulcers. Next day all the symptoms were worse, and he was leeched on the temples. Saline medicines with sulphate of magnesia—small quantities of wine and water. On the next day but one the patient died.

*Dissection.* The alimentary canal was laid entirely open, when it was discovered that the large intestines were in a state of extensive disease. "They were studded, more or less, nearly throughout their extent, with gangrenous ulcers, mostly of the size of an ordinary split pea." The peritoneal covering was sound, but the ulcerations had destroyed the mucous and muscular coats of the bowel. The bag of the cæcum presented one uninterrupted aspect of gangrenous ulceration; and, on sponging the parts, spots of mortification were seen, the intervening portions of mucous and muscular tunics being almost destroyed. The ascending colon was thickly studded with ulcerations, about three lines apart from each other. The transverse colon was also ulcerated, but the ulcers were thinly scattered. The descending colon and sigmoid flexures were nearly exempt. The other viscera of the abdomen and thorax were sound. The arachnoid was opake, and the pia mater partially infiltrated, and its vessels congested. The substance of the brain was firmer than usual; and rather more than the natural quantity of fluid was found in the ventricles.—*Lancet, No.* 203.

*Remarks.* Some degree of censure is conveyed by implication on the physician of the Fever Hospital, who sent this patient to Bartholomew's for the treatment of syphilis. Our knowledge of the talents and sagacity of Dr. Tweedie would induce us to conclude that there was some error in the report. Even if syphilis had been unequivocal, the state of fever in which the patient was, when admitted into Bartholomew's, precluded all idea of the application of specific treatment to that part of the complaint. But the state of the intestines, as discovered on dissection, was precisely that condition which cuts off nine in ten of those who go through the fever and die afterwards, without apparently adequate cause. We have brought forward several instances of this kind, and shall not cease to urge the attention of our brethren to this important investigation.

### 19. PNEUMONIA MISTAKEN FOR HYDROTHORAX.

[Drs. Graves and Stokes—Meath Hospital.]

*Case.* Dec. 1826, Bridget Hare, aged 35 years, was admitted, having been ill ten days, during which she took purgatives. Three days prior to admission, she had intense stitches in her sides, which disappeared when admitted. She has now dropsical swellings of the lower extremities—livid lips—swollen face—suffused eyes. She speaks in a loud whisper—has convulsive movement of the muscles of the face—is costive—belly slightly swelled—short cough—no expectoration—hurried respiration. "*Crepitus over the whole of both lungs, particularly about the right mamma, where the respiratory murmur is nearly inaudible. Along with a crepitous, in the left lung, is an acute sonorous râle.*" Percussion elicits a dull sound every where, particularly in the inferior part of right lung. The heart's action is strong through the stethoscope, and heard over an extensive surface—pulse 112 and soft—skin dry and hot—tongue white and moist—no vomiting or tenderness of the abdomen. Bled to 25 ounces. After bleeding, the respiratory murmur became more evident in some places, and the crepitus more moist. Thirty-six leeches were applied to the side, and a blister to the sternum. Took a mixture containing full four grains of emetic tartar, without vomiting. Two other venesections were employed, with additional blisters, and various other remedies; but the pulmonary affection had gained too much head, and she sunk on the 9th of December.

*Dissection.* Right lung universally adherent to the parietes—left apparently healthy—superior portion of right lung inflamed, nearly to hepatization. In this lung some tubercles were also found, and the lower portion was almost completely hepatized, with one spot in which grumous blood was diffused, forming what the French term "pulmonary apoplexy." The bronchial membrane was highly inflamed.

*Remarks.* "Here we see dropsical swellings arising in a person of 35 years of age and healthy habit, from cold. Although the acute nature of the disease and the well-marked pectoral distress should have directed the attention of the practitioner who attended her before her admission, *to the chest*, yet the treatment consisted merely in the exhibition of purgatives. The pneumonia was here overlooked, and the disease considered to be hydrothorax—a frequent and dangerous error. This patient's life would probably have been saved by the early use of the lancet. When admitted, the *stethoscope* pointed out a pneumonia almost general."—*Clinical Reports*, *p*. 18, 21.

---

### 20. DISEASES OF THE BRAIN AND SPINAL MARROW.

[Meath Hospital.]

The following cases are from the clinical practice of Drs. Graves and Stokes.

*Case* 1. HEMIPLEGIA. John Toole, aged 55, was admitted in the latter end of July, with the following symptoms:—hot skin, strong pulse, flushed face, head-ache, delirium. For these symptoms venesection and purgation were directed. A few nights after his entrance into hospital, he suddenly fell down, (while getting out to the commode) and remained senseless for a short time. On recovering, he was found to be hemiplegiac on the left side, with his tongue drawn to the *paralytic* side. Rigid diet and antiphlogistics were employed for a fortnight, without benefit. Strychnine was then given for a considerable time, without much appearance of operation. Tartar emetic was applied along the spine—an issue was inserted in the loins. At length he was able to stand a little, but attempted to walk with extreme difficulty. Has lately taken full diet, with a pint of porter daily. He labours under frequent agitation of mind—generally bursting into tears when asked how he is; though he always reports himself better. The strychnine was again resorted to, but with little advantage. Discharged incurable.

*Remarks.* The authors suppose, with reason, that during the febrile excitement, an effusion of blood took place into the right hemisphere of the brain. But how do they account for the tongue being drawn to the paralyzed side? This is contrary to the usual phenomenon in such cases. It is highly probable that the brain is softening round the effused clot of blood, and that a fresh attack of apoplexy will take place, and terminate the patient's life.

*Case* 2. Catharine Mooney, aged seven years, was admitted on the 7th October, having laboured for six weeks previously under paralysis of both lower extremities, passing her fæces and urine involuntarily. The complaint was supposed to have been brought on by lying in a damp bed for some nights. *The knees are drawn up, and any attempt to extend the legs gives great pain.* When supported, she can move the lower extremities, but cannot bear the weight of the body on them. She took small quantities of oil of turpentine internally, and had the same applied to the spine, with blisters to different parts of the back,

The strychnine was then given twice a day, and pushed as far as was considered prudent, but without benefit. All other remedies also failed, and the poor girl was dismissed unrelieved.

*Remarks.* We doubt not there is organic affection of the spinal marrow or its coverings in this case. The contraction of the paralytic limbs, with pain on attempts to move them, is not a very uncommon phenomenon in some affections of the brain, especially softening of that organ.

*Case 3. Colica Pictonum.* Michael Smith, aged 22, a painter, experienced, about two years ago, an attack of colica pictonum, which lasted a fortnight. During the last year, has had several slight attacks. After an attack, four months ago, he began to experience a difficulty of extending the index finger of the right hand. This was succeeded by pains in the arms and hands, with numbness and loss of feeling and power, first in the right hand, and afterwards in the left. The weakness at length amounted to inability to elevate the arm. He had some pain in the knee, but no considerable loss of power. The muscles of the arms are flaccid, and the whole member appears much extenuated. The pulse is 90, and of good strength—no belly-ache—good appetite —no cough, palpitation, or head-ache. Splints were applied to the fore-arm—warm bath—strychnine. The power of the hands and arms soon increased, and he observed that he had some subsultus in the palms of his hands, his fingers, and his wrists, with sensation of increased warmth and tingling in his fingers. Soon after this he began to feel a tingling in the lower extremities, and the power of motion in the wrists and fore-arms increased. The quantity of strychnine was decreased. Slight head-ache and dimness of sight induced the physicians to omit the strychnine for a time, after which it was resumed, and again the twitchings recurred. Ultimately he was discharged with nearly entire recovery of power and flesh.

*Remarks.* The strychnine was persevered in for nearly three weeks. "It evidently produced some of the symptoms mentioned by Magendie, such as tingling, increased warmth, twitches, or subsultus, and shooting pains in the paralytic limbs." At first it seemed productive of benefit, but as this was not progressive in proportion to the quantity taken, the physicians desisted from its use. The application of splints, with blisters to the nape of the neck and spine, seemed very useful. "The difficulty of extending the index finger of the right hand, the numbness and pains which preceeded the appearance of paralysis in the hands and arms, are worthy of notice. Similar phenomena are observed to precede paralysis depending on chronic affections of the brain and spinal marrow."—*Clinical Reports by Dr. Graves and Dr. Stokes.*

21. FRACTURE OF THE CRANIUM AND SPINE.

[St. George's Hospital.]

James Collins, æt. 32, a labourer, was precipitated from a height of 20 feet, with a heavy piece of iron, which fell across his back when he reached the ground. This was on the 4th July. On admission into the hospital, under Mr. Jeffreys, shortly afterwards, there was discovered compound fracture of the skull, on the right side, extending in a direction from the mastoid process to the sagittal suture. The bone was not at all shattered, and but slightly depressed. Besides this, the 8th or 9th dorsal vertebra was found to be broken, and the corresponding rib seemed to be fractured near its head. The parts below the fracture were paralysed—he moaned incessantly, and seemed to suffer much from his back, but there were no symptoms of compression. *July 5th.* The penis is in a state of semi-priapism—pain on pressing the back—pupils sluggish—moans and talks incoherently—has had no motion—pulse 92, rather full. *Urine drawn off—V. S. ad* ℥xx—*haust. sennæ 4tis horis dum disjt. alvus. Enema vesp.* *6th.* The blood drawn is not inflamed—has had several involuntary motions, and vomited, last evening, much undigested food and bile. He is not so stupid to-day—pulse 80, full and hard—tongue dry. Urine drawn off; it is of a dark colour and reddens litmus paper. *V. S. ad* ℥xvj—*h. sal. magn. sulph.* ʒss. *6tis horis.* In the evening he complained of pain in the sternum, and a fracture of the xiphoid cartilage was discovered. *Chest swathed with a flannel roller.* *7th.* Rather easier to-day. *9th.* Not so well to-day—the urine is ammoniacal for the first time—pain about the xiphoid cartilage—the respiration is, and has been all along, carried on chiefly by the diaphragm. *V. S. ad* ℥xij. *10th.* Much the same—blood not inflamed. *11th.* Hiccup in the night and this morning, occurring about every fifth inspiration—he lies in a stupid, half-insensible state, but when roused, answers questions, and continues to mutter and repeat the answer almost unconsciously, for some time. No motion or sensation below the fracture—stools loose—urine ammoniacal and bloody. *12th.* He is evidently sinking—the hiccup is very distressing—he vomited much brown fluid in the night and this morning. Pulse 100, small and weak. *14th.* Died this morning.

*Dissection.* The fracture of the skull was situated in the right parietal bone, about two inches in length, and stretching backwards and downwards towards its posterior inferior angle. It was not comminuted, and the depression no greater than a line or two. On raising the skull-cap, the inner table was found to be depressed, full a quarter of an inch or more, and to have penetrated the dura mater and cortical substance of the brain. There was no extravasation of blood between the bone and dura mater, but there was some effused beneath that membrane, particularly on the opposite side of the head; no fracture, however, or counter-fissure could be discovered on that side. Beneath the tentorium, and lying on the cerebellum, the quantity of extravasated blood was very considerable. No appearance of inflammation either of the brain or its membranes. On laying open the spinal canal, there was seen some extravasation upon the sheath. The body of the 9th dorsal vertebra was broken obliquely across, and its transverse process fractured off. The part of the column above the fracture, with the upper portion of the fractured bone had rather fallen forwards, whilst the lower part of the column inclined backwards, the lower portion of the broken bone thus projecting into the canal, and forming with its jagged edge, an angle, over which the spinal marrow was stretched. On making a section of the medulla, it was found to be, just at this point, of a reddish-brown colour, and soft bouille-like consistence, and on passing the finger over it, there was evidently some slight loss of substance. The 9th rib was broken at its spinal end. The abdomen and chest were but cursorily examined. Nothing particular could be observed about them.

*Remarks.* Such cases as this are not so interesting, practically speaking, as those which are more under the dominion of our art, and come home to us every day. At the same time they should not be passed over in silence, because, as far as we yet know, they are irremediable. The operation of trephining the spine would have been in this case, as we fear it would be in the great majority, worse than useless, for it was the body of the bone which was fractured. The patient died, apparently, more from the shock, than from the secondary consequences of the accident—irritation and inflammation. From the first, he never fairly rallied, but lay in a stupid dozing state, bearing a great analogy to that of concussion, save that here the concussion was not of the brain alone, but likewise of the spinal marrow and whole nervous system.

### 22. HEPATIC TUMOURS.

[M. Luroth. Strasbourg Hospital.]

*Case.* Peter Vacher, a shoemaker, aged 52 years, entered the hospital on the 14th January, 1824, having, for six years previously, perceived a tumour in the epigastric region, which, however, gave him but little inconvenience till about six weeks before the above date, when the tumour became painful and tender, accompanied by head-ache and much perspiration about the head. When examined, a circumscribed tumour was felt in the epigastric region, its site and the jaundiced appearance of the countenance leaving no doubt that it was hepatic. Yet the digestive functions were performed tolerably well—there was appetite—the tongue and pulse were natural—the bowels rather torpid. The patient was troubled with eructations, sleeplessness, head-aches. Laxative lavements were prescribed, and ox's gall was given, with the view of proving a substitute for the natural bile. It may readily be supposed that this remedy was of little service. The pain increasing, leeches were applied, followed by poultices. These means procured temporary relief; but the disease made progress, and the patient was worn out by the 4th March—the 50th day of his sojourn in the hospital.

*Dissection.* Both lobes of the liver were considerably enlarged, the left adhering to the spleen, and both lobes adhering to various surrounding parts, especially to the adjacent intestines. The *right* lobe of the liver was still more enlarged than the *left*, and when cut into, disclosed a cavity containing nearly a pint of a yellowish glairy, but clear fluid, in which several pieces of skinny membranes floated, evidently the debris of hydatid cysts. The cavity itself was lined by a cyst consisting of a dense white shining membrane, intimately adhering to the surrounding structure of the liver. The cavity of this cyst was intersected by partitions of a membranous structure. Around the cyst, in the substance of the liver, was found purulent matter, and also a number of tubercles, some of them softened down, some of them in a state of crudity. There was no other disease in the body.—*Repertoire, No.* 3, *p.* 53-4.

While translating the above, an interesting case occurred in our own practice, of which the following is a brief outline. A gentleman of very social habits, but by no means intemperate, had complained for some years of occasional uneasiness in the epigastrium, with slight symptoms of dyspepsia. Only three months before his death he was in his usual state of health, and presided at a public dinner. He had, however, an eruption on his face, which he was anxious to have removed, and for that purpose, put himself under the care of Mr. Macilwain and Mr. Brookes, of Lambeth. Mr. M. put the patient on a reduced scale of diet, consisting of a considerable proportion of vegetables, and gave some mild alterative and aperient medicines. By these means the eruption entirely disappeared from the face; but now a new train of symptoms occurred. Some fulness appeared just under the ensiform cartilage, attended with pain, and dyspeptic phenomena. For these symptoms, leeches were applied, and the usual remedies prescribed. But the fulness quickly increased, and Dr. Cholmely's attendance was had, in addition to that of the two other medical gentlemen. The epigastric swelling, however, made rapid progress, and it was soon discovered that the edge of the liver could be clearly traced extending from one hypochondrium to the other, considerably below the margins of the ribs. The biliary secretion was now considerably deranged—the countenance was sometimes tinged yellow—a sickly sallowness took place—much gastric irritability prevailed—and emaciation advanced. On the 14th July, Dr. Johnson was called in, in addition, and this was about ten weeks from the first appearance of epigastric swelling. There was now a large prominent tumour in the epigastrium, and the liver could be felt as low as the umbilicus, extending from one side to the other, with hard rounded edges. It was conjectured now that there was some morbid growth, probably of an hydatid, tuberculated, or fungoid nature, independently of the general enlargement of the liver, which might account for the large rounded prominence in the centre. Nothing could, of course, be done, but lessen the gastric irritability by anodynes and effervescing medicines, while motions were procured by aperients. The prominent symptoms were now paroxysms of severe pain along and under the edges of the enlarged liver, which pain was considered by the medical attendants as seated in the bowels, and not in the tumour itself. The unfortunate patient could only lie on his right side—he had constant fever—thickly coated tongue—thirst—loss of all appetite—very morbid secretions from the bowels—and loaded urine. His countenance was indicative of visceral disease. Sir Astley Cooper was now added, in consultation; but, alas! the disease was beyond the reach of art! The patient lingered till the 29th July, when he rather suddenly expired.

Dr. Johnson and Mr. Brookes examined the body at eight o'clock the same evening. There was some serous effusion in the abdomen, which was principally occupied by an immense liver, or rather tumour of precisely the appearance of brain. Not a particle of the original natural structure of the organ could be discovered. All was a homogeneous encephaloid mass, in which neither vessel nor duct could be traced by the naked eye. This enormous encephaloid growth (for it could hardly be called liver) had pushed up the diaphragm before it, and reached to the very summit of the thorax on the right side, descending below the umbilicus. Extensive adhesions had glued it to the stomach, transverse arch of the colon, and other contiguous parts. In a groove on its concave surface was found the gall-bladder, containing a thin dark-coloured fluid bearing little resemblance to bile. There was no other disease worth mentioning.

*Remarks.* We have said that the patient was not *intemperate*, in the common acceptation of the word; but he was a *bon vivant*, though he never carried the indulgences of the table to inebriety. We do not, indeed, believe that this disease was at all dependent on any dietetic irregularity. The rapidity of growth in this tumour, shewed a *malignancy* of character very different from common enlargements of the liver. We forgot to mention that, about twelve months before his death, he had been thrown out of a gig, and severely bruised; but, from this accident, he very soon recovered, and enjoyed his usual state of health till within three months of the termination of life. He was of a very philosophic turn of mind, and, a few days before his death, he left commands with his executor that his body should be examined, if his medical attendants expressed any wish to that effect.

Was the eruption on the face any index of the morbid growth going on in the liver? Had the disappearance of this eruption any connexion with the rapid development of the hepatic disorganization which quickly ensued? These are questions which are easily asked, but not so easily answered.

### 23. PROTRACTED LACTATION.

[Dr. Morton—Royal Metropolitan Infirmary.]

Dr. M. has come to certain conclusions on this subject, founded on cases which have presented themselves to him chiefly in public practice. The conclusions are these: 1mo. That, if children are suckled for an undue length of time, *i. e.* beyond the period of nine or ten months, they will be liable, in consequence, to inflammation of the brain. This proposition is supported by seven cases of children affected with cephalitis, where the period of lactation had been considerably protracted.

2ndo. That the same effect will take place, where the milk is furnished beyond the above period to a child, although *that* child may not have been at the female's breast from the beginning. *This is supported by only one case.*

3tio. That if the disease in question be not developed at once by the said protracted lactation, a predisposition to cephalic diseases will be established. *Supported by eight examples.*

4to. That children too long suckled when taken ill with other diseases, are much more liable to suffer in the head than children reared in a different manner. *Supported by six cases.*

Dr. M. professes his ignorance of the manner in which protracted lactation produces the complaint; but thinks it probable that the affection of the head is consequent upon derangement in the chylopoietic viscera. This may result from the poorness or other bad quality of the milk, by which it is unfitted for proper nutrition. Although the number of examples adduced by Dr. Morton is too inconsiderable to ground any fixed principle on at present; yet the subject will excite the attention of others, and multiplied observation may confirm or refute individual experience.

---

### 24. INSIDIOUS ABDOMINAL INFLAMMATION.

[Drs. Graves and Stokes. Meath Hospital.]

*Case.* Mary M'Dermott, aged 18 years, was admitted on the 21st of November, labouring under the usual symptoms of the prevailing epidemic, but not at all severe, and only of a few days' duration. The next day she had some nausea, but no other symptom of abdominal affection. This was relieved by effervescing draughts. On the 23d she was much better, and took no medicine. She continued so till the 26th, when she complained of tenderness in the epigastrium, with nausea, and quick, but not hard pulse. This epigastric tenderness was so common in this fever, and so generally subsided after the crisis, that it excited little attention. Effervescing draughts were again given. 27th, There was great pain in the abdomen, *without tenderness.* Pulse quick, small, and easily compressible—bowels free. Twenty leeches to the abdomen, followed by a large poultice, and a grain of calomel and one of opium thrice a day. 28th, She was relieved by the bleeding, and the abdomen was free from tenderness on pressure. The nausea continues—bowels free. 29th, Had severe vomiting in the night in spite of effervescing draughts with tincture of opium. The belly is excessively tender on pressure—she is covered with cold sweats—pulse imperceptible—lies on one side, with the knees drawn up—constant vomiting. In the evening she expired.

*Dissection.* Peritoneum was universally inflamed—the omentum adhered to the intestines and presented black spots, which corresponded to small perforations in the lesser intestines—considerable quantity of purulent matter in the abdominal cavity—small intestines covered with a complete investment of coagulable lymph—towards the termination of the ileum there was a large perforation—peritoneum lining the abdominal muscles inflamed. The mucous membrane of the stomach and small intestines does not appear to have been carefully examined, which is very surprising; but we conclude that it must surely have been inflamed from what the authors say in their remarks.

*Remarks.* Our authors ask here, "was the disease peritonitis? certainly not." The peritonitis they maintain, only commenced after the escape of the intestinal contents into the cavity of the abdomen. This we believe was the case; for, on the 27th, although the patient complained of pain, yet there was no tenderness on pressure, which last did not come on till 24 hours before death, along with the other symptoms of intense peritonitis. "Previously to the occurrence of the perforations there was undoubtedly inflammation of the mucous membrane of the stomach and small intestines, as denoted by tenderness of the epigastrium, fever, vomiting, &c."

The following case bears on the same subject.

*Case* 2. Mary Philips, aged 25 years, was taken with the ordinary form of fever, in the latter part of November. In the beginning of December she was convalescent; but it was remarked that she gained neither appetite nor strength. She lay in bed without making any complaint but that of weakness, and without any remarkable symptom, except that of an occasional circumscribed flush on the cheek. She had some nausea, and her pulse continued quick and small. On the 9th December, the debility was increased. Some latent pneumonia was now discovered, and she died on the 13th of the same month.

*Dissection.* There was some inflammation in the lower part of the right lung. The mucous membrane of the stomach was soft, thickened, and presented some black spots towards the cardiac extremity, where it was easily torn. The first six inches of the duodenum were of a deep red colour; but the mucous membrane was firm, and presented a number of white external elevations. Although the red colour disappeared beyond this portion, the enlarged glands were still very evident. Their number decreased till the middle of the jejunum was gained, where they nearly disappeared. In the lower portion of ileum, the vascularity again appeared, together with a number of enlarged mucous glands. The vascularity increasing, they discovered, about nine inches above the caput coli, flat circular ulcerations, about the size of a sixpence, of a yellow colour, with raised and hardened edges. Just above the valve of the colon there were numerous smaller ulcerations, of an irregular form, many of which had perforated the mucous coat of the bowel. The large intestines were healthy—the mesenteric glands enlarged.

*Remarks.* Our authors consider the white spherical elevations as mucous follicles morbidly enlarged—and this enlargement, probably the effect of inflammation. These appearances would be familiar to the eye of the English pathologist, if the internal surface of the intestines was properly examined in *post mortem* investigations.

---

### 25. EMPYEMA.*

[Mr. Johnson. Military Hospital.]

This case is calculated to excite some surprise: But we shall lay the particulars before our readers before we make any comments.

*Case.* A man, aged 26, was admitted into the Dublin Hospital, on the 29th March, 1826, with pneumonic symptoms, having been discharged ten days previously for a similar attack. He was bled ad deliquium. In the afternoon he was again largely bled—blisters were applied to the chest—and the antiphlogistic regimen enjoined. On the succeeding day he was bled for the third time, and the blood was highly inflamed all these times. On the 31st, or third day, there was a subsidence of the inflammatory symptoms, and digitalis was then administered. His cough continuing, and other symptoms indicative of phthisis, he was sent to his native place on the 8th of August. On the 5th October of the same year, we find the patient returned, and his general health apparently good. He was consequently sent to duty. This duty he continued to perform up to the 8th of April, 1827, when he was again admitted into the Regimental Hospital in London. He had now great oppression at the chest, with cough—shortness of breath—*quick undulating pulse*—white tongue—and "subject to pulmonic attacks." He was ordered to be bled largely. On the 11th a blister was applied to the chest. 26th, The breathing being again laborious, "bleeding became again indispensible." On the 14th May, he is stated to be anasarcous. The countenance became swollen and cachectic, and the neck, axilla, thorax, and lower extremities became infiltrated. "The stethoscope was applied several times to the chest, without any practical inference being drawn from these trials."

"He continued to sit up a few hours daily, until the last fortnight of his existence. Till then his state was comparatively easy, as long as he abstained from making any particular exertion: throughout his cough was not very urgent; expectoration not by any means profuse, and considered only muco-purulent. He invariably lay on the left side: he stated, that when in any other position his breathing became impeded. Towards the end he was particularly desirous of keeping his head low. I was called to visit him on the morning of the 28th of May, and found him in the last extremity, suffering greatly from impeded respiration, which was short and hurried; pulse hardly perceptible; mental faculties clear and coherent. It became necessary to lower his head, other-

---

* Med. Repository. August, 1827.

wise suffocation must have ensued: he expired two hours afterwards, at six o'clock in the morning." 106.

Mr. Johnson observes that he had omitted to mention that—" a few days before death a painful elevation of the integuments appeared under the left mamma, which, conveying a sense of fluctuation to the touch, a lancet was introduced, but no matter escaped."

*Dissection.* " The examination commenced from this point; and a cutting instrument having been passed into the former opening, a large gush of matter took place. As there could be no doubt of this opening communicating with the cavity of the thorax, it was closed, and the abdominal cavity was then examined. The liver was found occupying the middle region of the belly; its thin edge extending as low as the umbilicus. On the first superficial view, the impression was, that this viscus was preternaturally large: this, however, was not the case, as, on examination, it was found healthy in structure, with the exception of being paler than natural. On looking into the left hypochondrium, the appearance was in some degree accounted for,—as a large tumour presented, which had the effect of pushing the stomach down towards the umbilicus, and in its descent bringing (through the medium of its connexions) the liver and spleen with it.

" There was a good deal of serosity in the abdominal cavity. An opening was now made from the left hypochondrium, passing through the most prominent part of the tumour, dividing the diaphragm and pleura into the cavity of the chest: there was instantly a free escape of fetid matter, of the consistence of thin cream. From this opening, twenty-seven pints of fluid were taken, besides several pounds which escaped into the belly during the operation. The sternum was now raised, and the left division of the thorax freely washed out. The lung of that side was completely destroyed: there was merely a condensed substance at its root, where the branching-off of the pulmonary artery took place. The heart was completely pushed into the right cavity of the thorax; no part of it discernible to the left of the middle line of the sternum: it had lost all its obliquity of position, and gave an appearance of original transposition. In fact, its situation was perpendicular, and appeared *twisted* over, as the left auricle was the most anterior, or the first which presented. The pericardium was completely filled with water, and the heart of a pale colour. The right cavities of the heart were found empty; and beyond that, there was nothing unusual." 107.

*Remarks.* We cannot help noticing the observation which is made respecting the application of the stethoscope in the above case. Mr. Johnson only saw the patient the day he died, and therefore our remarks do not apply to him. But this we will say, that he who employed auscultation where 30 pints of matter were collected in the chest, *without being able to draw any practical inference*, might just as well have applied the butt end of the soldier's musket to the chest, and looked through the touch-hole. He could have known nothing of the *use* of the stethoscope, and therefore should not have spoken of it in terms of *abuse*. The operation which was performed on the fluctuating elevation under the left mamma, in the living body, forms a curious contrast with that which was performed after death!

---

### 26 SCIRRHOUS PANCREAS—CATARACT.

[M. King. Hotel Dieu.]

*Case*. Nicholas Dantieu was received into the Hotel Dieu, of Paris, on the 15th December, 1825, being then in his 45th year. He was affected with cataract of both eyes, of some years standing, which rendered him nearly blind. His skin was tinged of a greenish yellow colour all over, as well as his eyes. This icterus came on, according to his account, four months previously, from grief at the loss of sight. M. Dupuytren considering the jaundice as the effect of chagrin, and not the consequence of any internal disease, operated for the cataract of the right eye, by couching, and put the patient on low diet. The patient was partially restored to sight, and appeared to be going on pretty well; but about ten or twelve days after the operation he began to complain of great debility, tinnitus aurium, and tendency to syncope, when he attempted the perpendicular posture. By the 3d of January, 1826, these symptoms had considerably increased—he passed involuntary stools of black and bloody appearance, and next day he expired.

*Dissection*. The emaciation was moderate, the skin still of a greenish yellow colour—brain and membranes sound—stomach contracted and empty, with some very red spots on its mucous membrane. The duodenum was filled with reddish-coloured bilious matters, and its mucous membrane evidently inflamed. The internal surface of the small intestines was of a deep red colour, but the vessels were not injected—consequently the colour was considered as the effect of imbibition. The large intestines contained coagulated blood in a state of decomposition, and their mucous membrane was red as blood. The liver was large, and of a slate colour externally; but its interior texture was green. The gall-bladder, besides gas, contained some inodorous, tasteless, and colourless sero-mucous fluid, bearing no resemblance to bile. The cystic duct was free. The hepatic and choledoch canals were distended to the size of the ileum of an infant, and filled with gas. The mouth of the ductus communis was pervious; but the dilatation of these canals was evidently owing to the obstruction offered to the discharge of bile by the pancreas and other scirrhous glands. The pancreas was very large, and a portion of it scirrhous and amalgamated with a cluster of scirrhous glands situated between the pancreas and aorta, by which the biliary duct was compressed. The pancreatic duct was free. The heart was large and covered with fat. There was no other lesion of any consequence in the body. The fluid found in the gall-bladder was submitted to chemical analysis by M. Chevalier, but the results were not very satisfactory. At all events, it had no resemblance in sensible properties or chemical composition to bile.—*Repertoire, No. 3.*

The author hazards some speculations on the probability that the cataracts were occasioned by the disorder of the digestive organs resulting from the obstruction offered to the bile in its passage into the intestines. These, of course, are merely hypothetical. The case, however, is interesting, as shewing the dreadful effects of obstruction to the discharge of bile into the intestines. Not only was the secreting organ changed in structure from the retention of the bile; but the digestive apparatus and the whole system suffered from the want of the biliary fluid, so necessary to digestion.

---

### 27. PATHOLOGY OF FEVER.

[Dr. Hewett. St. George's Hospital.]

In the Medical and Physical Journal for August, Dr. Hewett has again presented the public with some interesting documents respecting the alterations of structure found as *consequences*—or, as some will say, as *causes* of fever. The report embraces a year at the hospital above-mentioned, viz. from the 1st. July, 1826, to the 1st. July, 1827. In that time, 190 cases of idiopathic continued fever were admitted, of which number 26 died, and 165 were discharged cured. A large proportion of the *former* were admitted in those advanced stages, when irremediable organic disease had taken place. So much depends on the period of admission in fevers, that we have never attached much importance to those favourable or unfavourable *numerical* results, which some institutions present, as compared with others. We happen to know that some little management—we had almost said manœuvering is employed in some quarters, which not a little influences the *apparent* ratio of mortality. But of that, it is not our hint to speak, at the present time.

In Dr. Hewett's report, a tabular view of the fatal cases is given, specifying the names of the patients, the duration of the fever, and the organs found affected on dissection. On examining these tables, one cannot help observing the predominance of diseased structure in the intestines, especially in the mucous membrane, as compared with lesions in the head and in the thorax. Still, the disproportion is not so great as to disturb the rational view which is now generally taken of fever in this country—namely, that the ravages of this disease are not confined to this or that organ, but fall, we might almost say, indiscriminately on one or other, according to circumstances, the exact nature of which we cannot always detect. Doubtless there are few individuals in whom all the great organs of the body are equally disposed or indisposed to disease. Hence, in fever, one organ is generally found to have borne a greater onus of disease than the others.

The most frequent species of lesion in the intestines (particularly the small intestines) was the follicular ulceration, described by Bretonneau and other French writers, as well as by Dr. H. himself, on a former occasion.

"That these follicular ulcers of the small intestines (seen in the above report to have so often occurred,) arise spontaneously during the progress of idiopathic fever, has been already sufficiently proved, and, when once established, it will be readily admitted that, though originally the effect of fever, they must (at least during their active and irritable state) reciprocally exasperate the symptoms, and become a very effective cause of the prolongation of fever; but the doctrine that their existence, when they have lost their irritability, and assumed a tranquil condition, is not incompatible with the subsidence of the fever, and even with some semblance of convalescence, may not gain so ready a belief, and may possibly require some further proof than the mere assertion of the writer." 112.

Two or three cases are detailed by our very intelligent author, which leave no doubt that a considerable degree of convalescence may take place, when these intestinal ulcers have lost their irritability, and thus the patient and practitioner are thrown off their guard, till some error in diet or other circumstance renews the irritability of the ulcers, and kindles up a new fever more dangerous than, and probably very different in kind from, the original.

In St. George's Hospital, as in all other parts of the kingdom, a considerable number of intermittents were seen, and all these were cured. Several of the continued fevers also changed into the intermittent or remittent types—probably connected, in some cases, with the state of the intestinal ulcers.

In two or three cases the ulcers had perforated the coats of the intestines, and violent peritoneal inflammation was consequently superinduced by the escape of the contents of the bowels into the general cavity of the abdomen. These accidents are often accompanied by signs which sufficiently indicate their occurrence, and enable the practitioner to prognosticate the issue.

In a case in private practice, Dr. H. ascertained the time which elapsed between the complete perforation of the intestine and the death of the patient.

The gentleman was attacked with fever on the 15th March, 1827, and was seen by Dr. Hewett on the 21st. He did not then acknowledge any pain—his skin was dry and harsh, not very hot—pulse 104, and feeble—abdomen not the least tender, but it was large and hard—tongue pasty and brown. He had experienced several rigors since the commencement of the fever, which were generally followed by heat and some delirium at night. Two grains of calomel, with one of ipecacuan, and a quarter of a grain of opium were ordered every four hours. He continued much the same during the four following days, the medicines being continued. On the 27th the pill was ordered to be taken only once in six hours, and small doses of castor oil were directed when the bowels were torpid. On the 28th blisters were applied behind the ears, and cold lotions to the head. On Dr. Hewett's visit, 30th March, the patient reported himself free from pain; but on pressing the abdomen, his countenance bespoke the utmost agony. The last motion voided was found to consist almost entirely of blood, which was never the case before. All hope of recovery had now vanished, and anodynes only were exhibited. He died on the following day.

*Dissection.* The vessels of the brain were rather loaded, and there was some serosity at the base of the skull. The mucous membrane of the lungs was sound. On opening the abdomen, the omentum was found feebly adhering to some of the convolutions of the intestines, at spots afterwards ascertained to be places where some of the follicular ulcerations had nearly perforated the peritoneum. In two or three places, the perforation had been complete, and a quantity of liquid fæces was found in the cavity of the pelvis. The largest perforation was about four inches from the ileo-cæcal valve. There were numerous other ulcers, in various stages of their progress, extending throughout the whole line of the intestines. There were none in the cæcum or colon. These ulcers were not surrounded by any increased vascularity, and even the valvulæ conniventes, though in parts eroded by them, retained their natural hue. The mesenteric glands were enormously enlarged, some of them being an inch and a half in length. The liver and other abdominal viscera were healthy.

Dr. H. is far from wishing to attach any undue importance to these follicular ulcers, and is fully aware that they are the result of only one of the many disorganizing effects of fever. "But while the latter, from the alarming symptoms with which they are attended, immediately command the attention of the practitioner, the former often insidiously complete the work of destruction, without even exciting a suspicion of their existence."

Dr. Hewett is entitled to the thanks of his brethren, for drawing their attention to this interesting investigation.

---

### 27. DIABETES MELLITUS.

[M. Luroth. Strasburg Hospital.]

*Case.* J. B. Canyon, a soldier, aged 40 years, entered the Hospital of the Faculty of Medicine, of Strasburg, on the 27th December, 1824, having been affected with diabetes for three years previously. The cause was unknown; but the progress of the disease was rapid, and the patient was reduced to a state of marasmus. He had no pains in the region of the kidneys, nor could any lesion be discovered by external examination of the abdomen. The urine was copious and sweet—the appetite voracious—the thirst inextinguishable—tongue white and moist—pulse, temperature, and alvine excretions natural. The quantity of urine made each day amounted to upwards of 30 pints. Various remedies were used, without any impression being made on the complaint. The plan of Rollo was then tried. By the third day of this treatment, the quantity of urine was diminished to eight pints per diem, and a most abundant perspiration daily covered the surface. The qualities of the urine became nearly natural. But now the appetite failed, vomitings took place, constipation became obstinate, the thirst was still intense, febrile phenomena were developed, debility rapidly increased, and dyspnœa, with cough, were added to the other symptoms. The patient lingered out till the 10th March, 1825, seventy-two days from his entrance into hospital, and then expired.

*Dissection.* There was nothing remarkable in the intestinal canal, except a few discoloured patches in the mucous membrane, which, in texture, was sound. There was also a very small ulceration of one of the mucous follicles in the lower portion of ileum, near the valve of the colon. No apparent change in the liver, spleen, pancreas, or kidneys. The vessels of these last were strongly injected. The ureters were dilated, and the urinary bladder was very capacious. Its coats were thickened, and their vessels and nerves strongly developed. In the chest, the morbid phenomena far exceeded the symptoms during life. There was hydrothorax in the left side—hepatization of the left lung, and, in its upper portion, a large cavernous excavation—universal adhesion of the right lung to the side, but its structure sound—hydro-pericardium—aneurismal dilatation of the pulmonary artery, two inches in diameter. There was no lesion in the brain or its membranes. The blood was every where fluid in the vessels, and mixed with air.—REPERTOIRE, No. 3.

On several occasions, in this Journal, we have stated that, of many dissections which we have seen of diabetic patients, not one presented sound lungs. On examining the records of published cases, where dissection was practised, we could scarcely find a single instance where some disease of the lungs was not evident. These facts have long ago led us to believe that diabetes was dependent on pulmonary disease, and, consequently, on some defect in the sanguifactive process. It may be said, indeed, that all these pulmonary diseases occur daily, without producing diabetes. This objection is more specious than solid. The same may be made to many other pathological conclusions. The same organic disease in the brain will, in one individual, produce epilepsy—in another, apoplexy—in a third, paralysis—in a fourth, insanity, and so on. We cannot account for the variety of effects resulting from the same cause; but if we invariably find disease in the lungs on the examination of diabetic bodies, we may reasonably conclude that such are not mere coincidences, but are to be viewed in the light of cause and effect.

---

### 29. CEPHALALGIA—AGUE—TUBERCLES.

[Bicètre. M. Guerard.]

A young man, (aged 20 years) of feeble constitution, had become affected with tertian fever three months before the date of report, accompanied by cephalalgia frontalis and giddiness, coming on periodically, but by no means in accordance with the fever. He had been bled and blistered before he entered the hospital, but without relief to the cephalalgia. This last, however, gave way to a seton in the nucha, and blisters to the temples. In the course of a fortnight after his entrance into the hospital, he began to evince signs of imbecility—general weakness—want of power in the lower extremities—involuntary discharge of urine. In two months more a diarrhœa came on, and proved very obstinate, although the other functions of the body appeared natural, and the appetite was craving. The emaciation advanced very rapidly—the intellectual faculties became almost annihilated, and he died about four months after he came into the hospital.

*Dissection.* In the cerebrum, there was nothing particular to be observed, except a very little more serum in the ventricles than usual. But on the upper surface, and on the median line of the cerebellum, was discovered a tumour, an inch and a half in diameter, of a yellowish-white colour, and very firm consistence—in fact, it was a veritable tubercle, surrounded by a softening of the cerebellum to some small distance. There was no other disease in the head. The lungs were found studded with tubercles, and the peritoneum, both lining the parietes of the abdomen, and where it is reflected over the viscera, presented innumerable tubercles, of all sizes, from one to sixteen lines in diameter. Several of the intestinal folds had contracted adhesions, and the whole mucous membrane of the small intestines was covered with ulcerations of various sizes, many of them penetrating to the peritoneal coat.

The lesion discovered after death in the brain, is certainly not in accordance with the ideas generally entertained, respecting the functions of particular parts of the sensorium.

---

### 30. CLINICAL REPORT ON THE SEAT OF FEVER, BY M. BALLY.

[Cochin and La Pitié.]

The last quarter of 1826 furnishes the materials for this report, which is entirely dedicated to the paramount subject of investigation on the Continent—GASTRO-ENTERITIS. We too, deem it a subject of high interest; and are glad to see that it excites peculiar attention in this country, especially among hospital physicians.

It is, says our author, by connecting morbid phenomena with the local lesions on which they depend or are connected, that the moderns have arrived at exact knowledge of those grave and complex affections to which the ancients have applied the vague terms of putrid, malignant, nervous, and adynamic fevers, which are, in reality, veritable *gastro-enterites* degenerated, or as they are more properly and precisely termed by M. Bally, *ileo-diclidites*, signifying *inflammation of the ileum and upper surface of the valve of the colon*, the parts most commonly affected in fevers. M. Bally concludes, from a passage in Hippocrates, that the Father of Medicine was acquainted with this remarkable fact. He observes in the third book of his Epidemics, that, "among those individuals who perished, whether the disease ran a quick or a protracted course, the greater number died of affections of the bowels." This affection, M. Bally thinks, could be nothing else than inflammation. In examining the cases of fever detailed by various authors, and by Pinel among the rest, he considers it impossible not to conclude that the majority laboured under that state of the intestinal canal so well described by Broussais and his disciples. Those who have taken the pains to open many bodies after death by fever, afford innumerable proofs of the phenomenon in question, though they looked upon the disease as a concomitant or consequence rather than a cause of fever. In short, says he, if we compare the descriptions of putrid and malignant fevers, as handed down to us, with those of the gastro-enterites of the present day, we cannot but be struck with the resemblance. All allow that inflammation of the mucous membranes is a very common *attendant* on fever—and for his own part, M. Bally believes it to be the disease itself—or, at least, the first and principal lesion in fever.

The fevers of the last quarter of 1826 have tended to confirm M. Bally in the truth of this doctrine; and what is more, they have enabled him to make some distinctions not hitherto much noticed, and to adopt an important and successful method of treatment.

Scarcely had intermittents begun to subside, when inflammations of the digestive tube became prevalent. Some of them were hæmorrhagic, and terminated fatally in a very prompt manner. The greater number, however, assumed a low or typhoid type. These forms were more dangerous in proportion as they succeeded early to the intermittent epidemic; and M. Bally conceived that there was an intimate connexion between the two epidemics, especially as the gastro-enterites frequently assumed a quotidian intermittent character. When this was the case, the antiphlogistic treatment did not succeed, and the sulphate of quinine was effectual. The state of adynamia, which often supervened in the course, or towards the close of the fever, did not appear to result from any primitive debility of the subject, or as consecutive of gastro-enteritis; but it seemed often to depend on the lesion of some other organ, over which the antiphlogistic treatment had not effectual control. The rigidity of the limbs, the tremors, the subsultus tendinum, the throwing back of the head, the delirium, the ramblings, the loquacity, the difficulty of expanding the chest—all these functional disorders, says our author, were far from constituting the debility. They showed themselves during the existence of the most acute irritation, and they subsided with it under a very active system of depletion. They must, therefore, have depended on disorder of some particular organ.

M. Fodera considers the superior portion of the spinal marrow as the principal seat of affections truly adynamic; and our author partakes of the same opinion. He thinks this is confirmed both by experiments and by pathological anatomy. The above portion of spinal marrow has been often found softened in the dead body by our author, M. Bally. Stoll, whose authority is good on all points of practical observation, long ago remarked that putrid fevers did not always infer fevers of debility. He was somewhat astonished to find that bleeding and other evacuations cured the disease better than tonics. The oppression and the prostration of muscular power, although different in their nature, acknowledge the same cause. "The first results from congestion; the second, from inflammation of the spinal marrow." Bleeding, he observes, is more effectual in relieving the former than the latter condition of parts. "Thus, in the beginning of all adynamic fevers, the symptoms which constitute debility are owing to congestion or inflammation of the spinal marrow, either primitive or consecutive of gastro-enteritis." We agree with our author that great errors are daily committed by pathologists in considering that there can be no *disorder* in a part during life, if no trace of change of structure be discernible after death. The organization of the brain, nerves, and their investing membranes, is so very delicate, that disorder of function sufficient to destroy life every day occurs, without alteration of structure. Hence the necessity of taking into consideration the symptoms, as well as the *post mortem* investigations.

Numerous dissections, made with the greatest care, have convinced M. Bally that inflammation of the lower portion of the small intestine, and upper surface of the ileo-cæcal valve, constitutes the first period of what are termed putrid or typhous fevers. If this inflammation be checked by nature or art, the subsequent symptoms of debility or putrescency do not occur. If not, the disease proceeds to ulceration or destruction of the mucous membrane. It is very rare to find any alteration of structure in the duodenum, jejunum, or colon. The cæcum itself is seldom affected, although the upper surface of the valve is so generally implicated in the disease. What can be the cause of this locality of the disease in the part above-mentioned? M. Bally attributes it to the difference of acrimony in the contents of the small and of the large intestines. Nature, he says, is sometimes equal to the task of curing these ileo-cæcal inflammations—and, in one instance, this was effected by a profuse nasal hæmorrhage.

The division of the disease into two stages or periods, is not only philosophic, but of great importance in a therapeutic point of view. In the first period, or that of inflammation, the antiphlogistic treatment may arrest the progress of the disease, and prevent the degeneration of the fever into the putrid or ulcerative form. But this disease, though so formidable and fatal in its advanced stages, does yet commence in so insidious a manner, that the patient is not soon enough alarmed, and is seldom sent to an hospital till after the first or second week, when the local affection has made great head, and when the constitutional symptoms veil those of the primary topical affection. As soon as the symptoms indicate that the inflammation is passing into the state of ulceration, it is necessary to abandon the antiphlogistic treatment, as far as sanguineous depletion is concerned, and to have recourse to powerful counter-irritation, so as to attract to the surface that morbid excitement which is going on internally. M. Bally does not, however, employ blisters for the purpose of counter-irritation. He has not seen advantage arise from their application in ileo-diclidites—but, on the contrary, disadvantage, from the property which the lytta has of causing irritation in the internal tissues. Mustard cataplasms have produced much better effects in the way of revulsion, when the brain or other organ was sympathetically affected. But he seems to place his principal dependence on a plentiful crop of pustules, brought out by tartar-emetic in the umbilical region. He has never seen any reason to conclude that the antimony is absorbed on these occasions—a position which we ourselves can substantiate by wide experience of this powerful counter-irritant. M. Bally employs a tartar-emetic plaster rather than ointment, to the abdomen, and in this respect we also coincide with him. It is far less troublesome, and far more powerful than the unctuous frictions. He sometimes applies a dozen of leeches to the part, and as soon as these animals fall off, he places a Burgundy-pitch plaster, well powdered with tartrate of antimony, over the leech-bites. In less than 48 or 64 hours a plentiful crop of pustules is elicited, each pustule surrounded by an areola of inflammation. If any species of counter-irritation can relieve the internal malady, this will do the business. In very grave cases, M. Bally employs an antimoniated drink for several days in succession, with the view of copiously evacuating the acrimonious contents of the small intestines, which prove a powerful source of internal irritation.

"Finally," says he, "when quotidian exacerbations, analogous to the periodical accessions of an intermittent fever, become associated with the ileo-cæcal affection, the sulphate of quinine is to be exhibited, notwithstanding the redness of the tongue, the abdominal pains, the diarrhœa, and the various other signs of inflammation of the mucous membrane of the intestines."

Such was the practice pursued by M. Bally, and, in general, it was productive of the best effects. We shall now give the particulars of a few cases in illustration.

*Case* 1. Peter Fontier, aged 23 years, had been exposed to wet and cold while in a state of perspiration. On the following day he experienced chills, fever, nausea, pains in the bowels, and diarrhœa. He entered the Hospital Cochin on the second of November, the 21st day of the complaint. The following were the prominent phenomena:—decubitus dorsalis, stupor, flushed face and injected conjunctivæ, tongue dry and red at the sides, epigastrium painful on the least pressure, much diarrhœa, subsultus tendinum. *Prescribed lemonade with gum arabic.* *22d day.* He this day had an abundant discharge of black blood from his nose, amounting to 12 ounces. The head is now more free, the abdomen more supple and less painful, the pulse softer. *23d day.* Another epistaxis, though less abundant. The tongue is very red, the alvine evacuations copious, frequent cough, increase of subsultus tendinum. *24th and 25th days.* Nasal hæmorrhages each day, which do not appear to afford relief. The decubitus dorsalis is now complete, with occasional delirium. The lips and tongue are covered with black sordes—the abdomen is tense and distended, and he has ten or twelve motions daily. *Sinapisms to the abdomen. 26th day.* Another nasal hæmorrhage, and the tongue is less red at the sides, the purging more moderate. *27th day.* Epistaxis moderate—great debility—coma. *Lavements of decoctum cinchonæ—sinapisms renewed. 28th day.* The epistaxis did not take place—abdomen not painful—tongue begins to clean. From this time, the disease decreased daily and convalescence gradually ensued, with ultimate recovery.

*Case* 2. Bougerie, aged 25 years, had experienced, for several days, a bloody diarrhœa, with colicky pains, tenesmus, and pains in his head. He was bled largely from the arm, and 14 leeches had been applied to the anus, with evident relief. He entered La Pitié on the 18th October, the eighth day of his illness. He had then great prostration of strength, with disposition to delirium—flushed face—injected conjunctiva—dilated pupils—dry tongue, red at the sides—urgent thirst—abdomen supple, but swelled and painful on pressure—diarrhœa, with some blood in the stools—pulse hard and frequent. *Rice water with gum. 9th day.* Delirium—intense heat of skin. 30 *leeches to the anus. 10th day.* Slight epistaxis—dorsal supination—cheeks red—cephalalgia with delirium and subsultus tendinum—lips, teeth, and gums, fuliginous—tongue the same as before. Pressure on the umbilical region gave pain, as also in the right iliac region. The intestinal evacuations are suspended—dysury—pulse strong. *Sixty leeches to the abdomen, followed immediately by a large mustard cataplasm. 11th day.* The same prostration of strength; but the head is more free, the urine more copious, the tongue less red, and the abdomen less tender—pulse softer and slower. *12th and 13th days.* Considerable improvement—tongue yellow—several loose stools—skin natural. *14th day.* Several hours of tranquil sleep—some return of appetite. From this time the tongue began to clean, and convalescence was soon established.

*Case* 3. Julien, aged 24 years, entered the Hospital Cochin, on the 11th of December, the eighth day of his illness, which had commenced with chilliness and shiverings that lasted 48 hours, succeeded by cephalalgia, nausea, and bad taste in his mouth. *9th day.* The face is flushed—the conjunctivæ injected—cephalalgia—tongue swelled, red, and furred—thirst urgent—epigastrium painful on pressure—diarrhœa—breathing oppressed, pulse strong and full, skin hot. *Lemonade with gum*—40 *leeches to the anus—frictions. 10th day.* The tongue very red—abdominal pains acute—20 liquid evacuations from the bowels. *11th day.* Great agitation—patient complains much of his head—face flushed—delirium—lips and teeth fuliginous—sensibility of the abdomen exquisite—pulse very quick—some tremor of the upper extremities. Sixteen leeches to the umbilical region, followed by a tartar-emetic plaster over the bites. *13th day.* Same state of agitation—loquacity—dryness of the mouth—burning thirst—several liquid stools—swelling of the abdomen. *Emollient lavements. 14th day.* Last night was more calm; but the tongue is still red, and the pulse quick and sharp, with heat and dryness of skin. 15*th day*. The patient is better, and asks for food. All the symptoms are ameliorated. The plaster is taken off, and there are numerous large pustules where the leech-bites had been. From this time, convalescence regularly advanced, and the patient entirely recovered.

We must pass over some interesting cases, where the event was equally fortunate through the same means, in order to give some particulars of a case that proved fatal, and where dissection took place.

*Case* 4. Etienne, aged 27 years, entered the Hospital Cochin on the 11th of October, stating that he had been ill a fortnight. He had passed the whole of a day in a damp cellar occupied in hard labour, and from this he dated his illness, which commenced with shiverings, cephalalgia, pains in his limbs, &c.—the shiverings coming on regularly in the evenings. He had taken plenty of hot wine and bouillon. 16*th day*. Face flushed—tongue coated in the middle, and red at the sides—thirst urgent—abdomen not tender—diarrhœa—pulse high, hard, and frequent—skin covered with perspiration. *Lemonade with gum*—15 *grains of sulphate of quinine in the* 24 *hours*. 17*th*. The same accession of symptoms in the evening—the same treatment. 18*th*. Delirium was added to the evening paroxysm, with watery diarrhœa. 18 grains of sulphate of quinine. 19*th*. The evening accession was only known by a strong excitement of fever, the skin being moist. The sulphate of quinine suspended. 20*th day*. Tongue very dry and red—much alteration for the worse—pulse strong and frequent. 21*st day*. Delirium—decubitus dorsalis—nausea—pulse 90. 22*nd day*. Not much delirium, but great prostration of strength—tongue and lips fuliginous—abdomen distended. 23*d day*. Somnolency—head drawn backwards—subsultus tendinum—involuntary defæcation. The patient lingered on till the 38*th day*, when he became affected with tetanic rigidity throughout the whole body, and, in a few days more he expired.

*Dissection*. The colon and the abdomen, in general, were distended with gas. The mesenteric glands were enlarged and inflamed—coats of the stomach red, but not softened—duodenum contained a yellowish matter—internal surface of the ileum covered with innumerable ulcerations, of various sizes, but increasing in number as the valve of the colon was approached. The mucous membrane in the interstices was much thickened. The liver and spleen were enlarged. There were various traces of inflammation and effusion in the brain and its ventricles, as well as in the spinal marrow. The latter was softened for the space of two inches opposite the sixth cervical vertebra, and also there was a softening abreast of the first vertebra (counting from below) extending down towards the 8th dorsal vertebra. There was no other lesion of any consequence in any part of the body.

From the foregoing and many other cases too numerous to introduce, M. Bally considers himself borne out in the doctrine which he maintains—namely, that the peculiar inflammation and ulceration seen in these fevers is not a common gastro-enteritis—the stomach being rarely affected; and that only secondarily—but an *ileo-diclidite*, or affection of the ileum and superior surface of the valve of the colon. M. Bally dwells on the importance of recognising this pathology of the disease, if it be correct, as it will tend to restrain the routine practitioner from throwing in stimulants and tonics at an early stage of the disease, from dread of debility; and induce him to use proper depletion and cooling aperients, in order to prevent the ulcerative stage that may otherwise ensue. We recommend these observations to the attentive consideration of our brethren, so that they may be brought to the test of experience. As we said in the beginning of this paper, there is great reason to believe that these inflammations and ulcerations of the mucous membrane of the intestines, are much more common than practitioners are inclined to acknowledge—probably from want of that minute attention to post mortem examinations so conspicuous on the Continent.—*Journal Gen. de Med.*

### 31. DR. LEE ON NUTRITION OF THE FŒTUS.

Some investigations made by the above-mentioned ingenious and talented young physician would lead us to think that the liver secretes a fluid during utero-gestation, which mainly serves the purpose of nutrition in the fœtus. He ascertained that the stomach of the fœtus, from three to nine months old, always contains a transparent mucous and acid fluid, but never the smallest admixture of albuminous or nutritious matter—while, on the other hand, the upper half of the small intestines always contains a yellowish pultaceous mass which, in appearance and chemical properties, exactly resembles the chyme of the adult—in a word, pure albumen. The lower half of the small intestines contains very little of this substance. The meconium is confined solely to the large intestines. But the most remarkable fact is, that a fluid resembling that in the duodenum, viz. pure albumen, is found in the hepatic duct of the fœtus—whence it may be fairly inferred that the liver secretes the nutriment of the fœtus, which is taken up from the small intestines. If these facts be corroborated by future investigators, and we venture to predict that they will, we shall have a satisfactory explanation of the large size of the liver in the fœtus, and of the important effects which the bile, in all ages of the individual, produces on the animal economy, and particularly on the growth and support of the body. Dr. Lee was assisted in his chemical investigations by that profound and accomplished chemist, Dr. Prout. Dr. Lee stands a fair chance of being handed down as a discoverer, even in this advanced æra of physiology.

